# Public Documents Digitization: Towards Efficiency and Reduction of Municipal Expenses

**DOI:** 10.12688/f1000research.169678.1

**Published:** 2025-09-12

**Authors:** María Maricely Chuquilin Condor, Segundo Amado Cajusol Yovera, Víctor Hugo Puican Rodríguez

**Affiliations:** 1 LA LIBERTAD, Cesar Vallejo University, Trujillo, La Libertad, Peru

**Keywords:** Digitization, cost reduction, administrative efficiency, document security, administrative transparency.

## Abstract

**Background:**

Documentary digitization in municipalities has emerged as a critical strategy for enhancing efficiency in administrative processes and reducing operational costs, aligning with the objectives of Sustainable Development Goal 8, which promotes sustained, inclusive, and sustainable economic growth. In the context of Latin America, and particularly in Peru, municipalities face significant challenges related to bureaucracy, inefficient resource allocation, and limited technological adoption. Therefore, the implementation of document digitization represents not only a technological innovation but also a mechanism to strengthen transparency, accountability, and sustainable management practices within the public sector.

**Methodology:**

This study employed a quantitative approach with a non-experimental, descriptive, and explanatory design. The research was conducted in a municipality located in the Amazonas region of Peru. The target population consisted of 125 administrative workers, from which a statistically representative sample of 95 was selected. Data were collected through structured questionnaires and analysed using descriptive statistics and inferential techniques to assess the relationship between documentary digitization and cost reduction.

**Results:**

The findings revealed that the adoption of document digitization significantly contributes to improving resource management, minimizing the duplication of tasks, and reducing operational costs associated with paper-based procedures. The study also identified secondary benefits, such as enhanced information security and improved knowledge management practices, which collectively strengthen institutional performance.

**Conclusion:**

This research provides empirical evidence that document digitization exerts a significant influence on cost reduction and overall efficiency in municipal management. Beyond economic savings, the process enhances organizational sustainability by promoting secure information handling and fostering a culture of innovation within public administration. The study’s relevance lies in its demonstration of how digitization can optimize public sector performance, while its novelty is reflected in the practical application of these principles in a municipality in Amazonas, Peru.

## Introduction

Document digitization in municipalities has become a crucial topic in contemporary public administration, offering significant improvements in the efficiency of resource management, operational productivity, and service quality; the transition from physical to digital documentation and only facilitates faster access to information but also reduces operational costs by optimizing management processes; in municipalities in Amazonas, the digital transformation of administrative documents aligns directly with Sustainable Development Goal 8, focusing on promoting economic growth, decent work, and resource efficiency.

Despite technological advances, many local governments around the world continue to face high administrative costs and inefficiencies due to reliance on physical documents and traditional processes. Municipalities in several regions, including Amazonas, face similar problems of inadequate document management infrastructure, which hinders operational efficiency and inflates administrative costs; document digitisation has proven to be an essential solution to improve information accessibility, reduce storage costs and enable more effective decision-making ( Oliveira et al., 2021; Ramos, 2021; Akaaboune et al., 2020).

At the national level, several municipalities in Peru, including those in Amazonas, continue to face challenges in implementing digital systems due to problems such as lack of integration with existing infrastructure, insufficient technological investment and the absence of comprehensive regulatory frameworks; these barriers delay the modernisation of public services and limit the potential benefits of document digitisation, including cost reduction and improved public service delivery ( Culqui et al., 2021; Cabero-Almenara et al., 2024).

This study specifically examines the impact of document digitisation on cost reduction in a municipality in Amazonas; the research is motivated by the need to assess how this digital transition can improve the efficiency of public administration, reduce waste of resources and promote better service delivery to the public; the study focuses on identifying the challenges and potential benefits of document digitisation in a rural Peruvian context, contributing to the broader debate on the digital transformation of government.

### Literature review

Villegas-Cervantes and Velásquez-Gutiérrez (2023) the global trend towards digital transformation of public management underscores the need for municipalities to modernise their operations; local governments around the world are increasingly adopting digital technologies to streamline processes and improve transparency; however, many rural and less developed regions, such as Amazonas, remain at the forefront of these challenges, where technological infrastructure and digital literacy are still developing.

Recent studies show that municipalities often face significant inefficiencies in physical document management, leading to delays and higher operational costs; although digitisation of documents has been shown to reduce these inefficiencies, many local governments have not yet fully embraced these changes due to financial, technical and regulatory barriers; this research aims to fill the gap in understanding how digitisation can specifically impact municipal cost structures in Amazonas, with a focus on reducing waste, improving labour productivity and optimising overall resource management ( Hagen, 2023; Andersson et al., 2022).

Although there is significant research on the benefits of document digitisation in public administration, there is limited focus on its application within rural municipalities in developing countries such as Peru; most studies have focused on urban centres or regions with advanced technological infrastructures, leaving a gap in understanding the challenges and potential of digitisation in less developed regions such as Amazonas; furthermore, the lack of studies specifically examining the financial impact of document digitisation on municipal cost reduction in Amazonas further highlights the need for this research ( Espinoza et al., 2023; Broccardo et al., 2023).

This study contributes to the literature by focusing on the specific context of one municipality in Amazonas, Peru, and exploring the direct influence of document digitisation on reducing administrative costs and improving operational efficiency; by addressing this gap, the research provides valuable insights into the potential of digital transformation for public management in rural and resource-constrained areas.

This study to answer the following research questions:

1. What is the influence of document digitization on operational efficiency and cost reduction in a municipality of Amazonas, Peru?
2. How does the implementation of document digitization impact labor productivity and resource management in and how can these challenges be overcome?
3. What are the key challenges faced by municipalities in Amazonas when implementing document digitization and how can these challenges be overcome?

The main objectives of this research are:

1. To determine the influence of document digitization on operational efficiency and cost reduction in a municipality of Amazonas, Peru.
2. To explore how the transition from physical to digital documents improves resource management and labor productivity in local governments.
3. To identify the key challenges and barriers faced by municipalities in Amazonas regarding document digitization and propose solutions to address these challenges.

This study is significant as it addresses the gap in the literature on document digitisation in rural municipalities, particularly in the Peruvian Amazon; by analysing the specific impact of digital transformation on municipal cost structures, this research provides valuable information for policy makers, municipal administrators and researchers involved in public administration and digital governance; the findings of this study will inform strategies to improve operational efficiency, reduce costs and improve service delivery in municipalities with limited technological resources.

The main contribution of this study lies in providing empirical evidence on the relationship between document digitisation and cost reduction in public sector organisations, specifically in rural municipalities in Amazonas, Peru; it extends the theoretical framework of digital administration and organisational efficiency by offering insights on how rural municipalities can leverage digital technologies to modernise public administration practices and achieve better resource management.

## Methods

### Research approach and applied nature

This research is applied in nature, focusing on using the knowledge generated to address practical challenges related to document management and efficiency in public expenditure in a municipality; a quantitative research approach was employed, enabling the analysis of statistical data to understand the relationship between document digitization and cost reduction; this approach allowed the collection of numerical data, performed statistical analysis, and generalized the results from the selected sample.

### Research design and scope of the study

A non-experimental cross-sectional design was chosen because the research did not manipulate the independent variables, but rather observed and analyzed the variables as they occur at a given time; this design was adequate to describe and explain the current situation of document digitization and its influence on the municipality’s expenditures without intervening in the existing processes; the scope of the research was limited to the evaluation of the implementation of document digitization in a municipality in Amazonas, Peru, as a detailed understanding of how digitization improves administrative efficiency and reduce operating costs was achieved.

### Inductive nature of the research

Moreover, this research can be considered as inductive in nature as it starts from empirical evidence to build constructs that can contribute to solid theoretical bodies and evidence that contribute to the process of Kuhnian positive heuristics in the field of public administration studies ( Drakopoulos & Karayiannis, 2005; Bell et al., 2022).

### Components of the document digitalization variable

Figure 1 illustrates the components that make up the document digitisation variable, as follows Digital transformation and knowledge management, Digital transformation of administrative documents, Security of digital documents and Legal risks of digitization.

**Figure 1.**
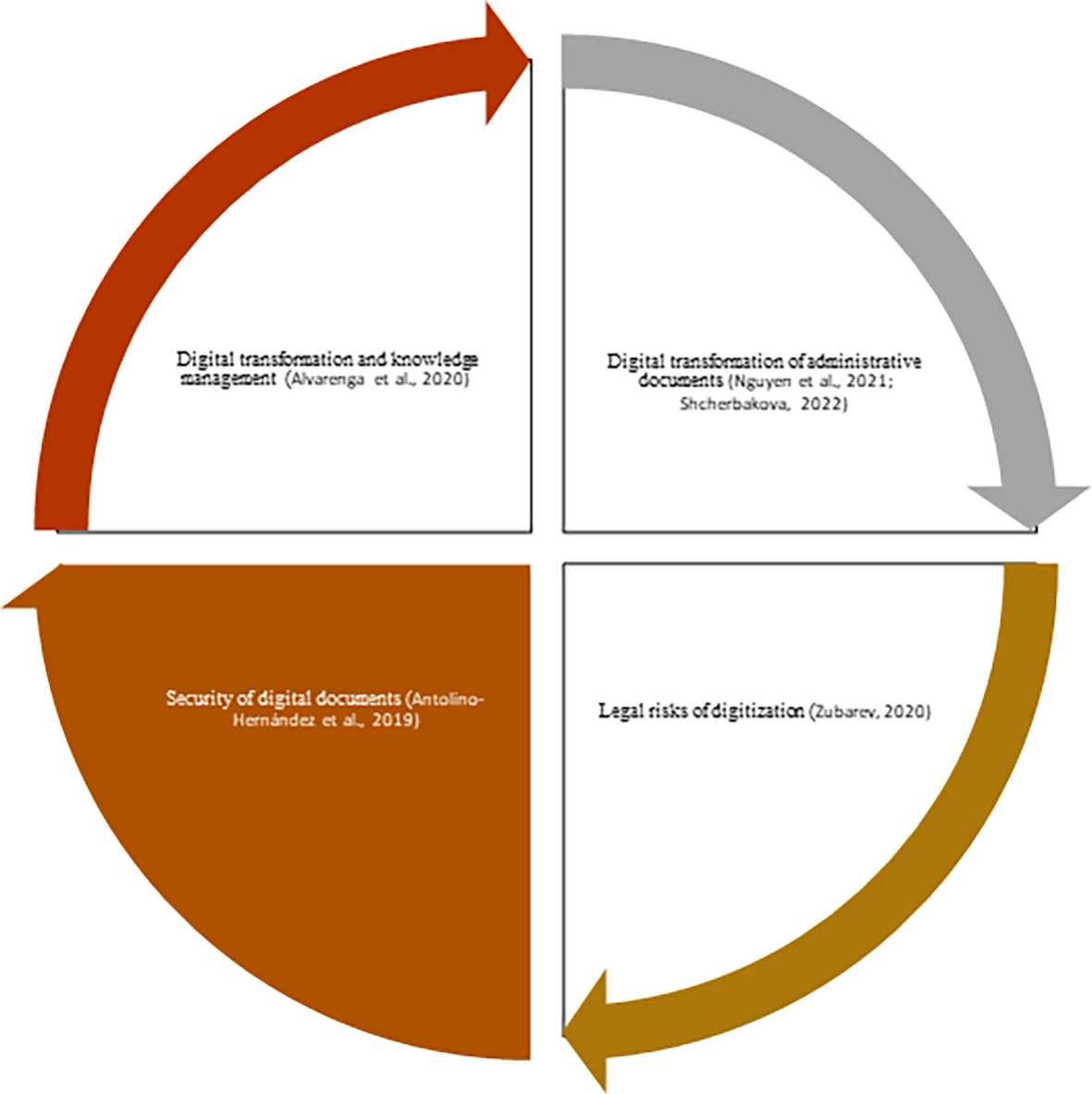
Components of document digitization. Note: *Components of document digitization.*

### Components of the cost reduction variable

Figure 2 illustrates the components that make up the cost reduction variable, as follows Expenditure quality, Use of resources efficiently and Effectiveness of resource use.

**Figure 2.**
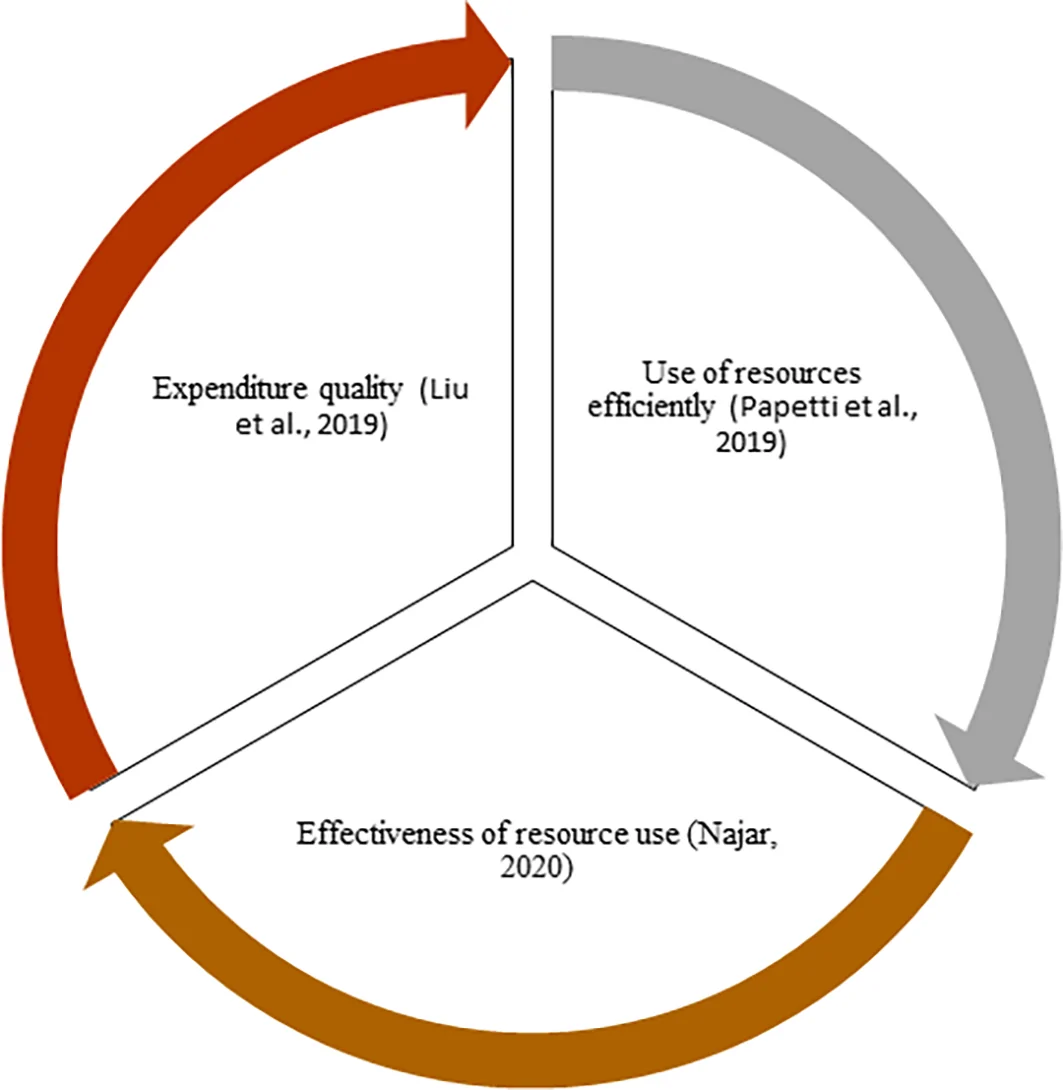
Components of cost reduction. Note: *Components of cost reduction* **.**

### Study population and sample

The study population consisted of 125 administrative workers from a municipality in Amazonas, Peru. Inclusion criteria required participants to have at least one year of service in the municipality, while those with less than one year or on probation were excluded. Using the finite proportions formula, a sample size of 95 administrative workers was determined as a representative sample. Stratified probability sampling was used to ensure a proportional representation of all administrative areas of the municipality, which increased the accuracy and validity of the results.

### Data collection and instrument

Data were collected through a survey using a 90-item questionnaire. The questionnaire was validated by experts with extensive experience in the field to ensure its validity and relevance. The reliability of the questionnaire was assessed using Cronbach’s alpha, with values of 0.912 for the first variable and 0.916 for the second. The survey was administered through face-to-face interviews, ensuring confidentiality and the voluntary participation of participants.

### Data analysis

Descriptive statistical techniques were used to summarise and organise the data, highlighting the main characteristics of the variables studied. Inferential analyses were also performed to test hypotheses and determine relationships between variables, allowing inferences to be drawn from the sample to the wider population. Statistical analysis was carried out using SPSS v27.

Ethical considerations were strictly adhered to throughout the research. Participants’ personal data were kept confidential and informed consent was obtained from all participants, ensuring that they were fully aware of the aims of the research and of their right to withdraw at any time without negative consequences.

## Results and discussion

### Results

Figure 3 illustrates the level of digitisation of documents in the municipality of Amazonas, Peru; the data reveals that most respondents reported moderate to high levels of adoption of digitisation in their administrative processes.

**Figure 3.**
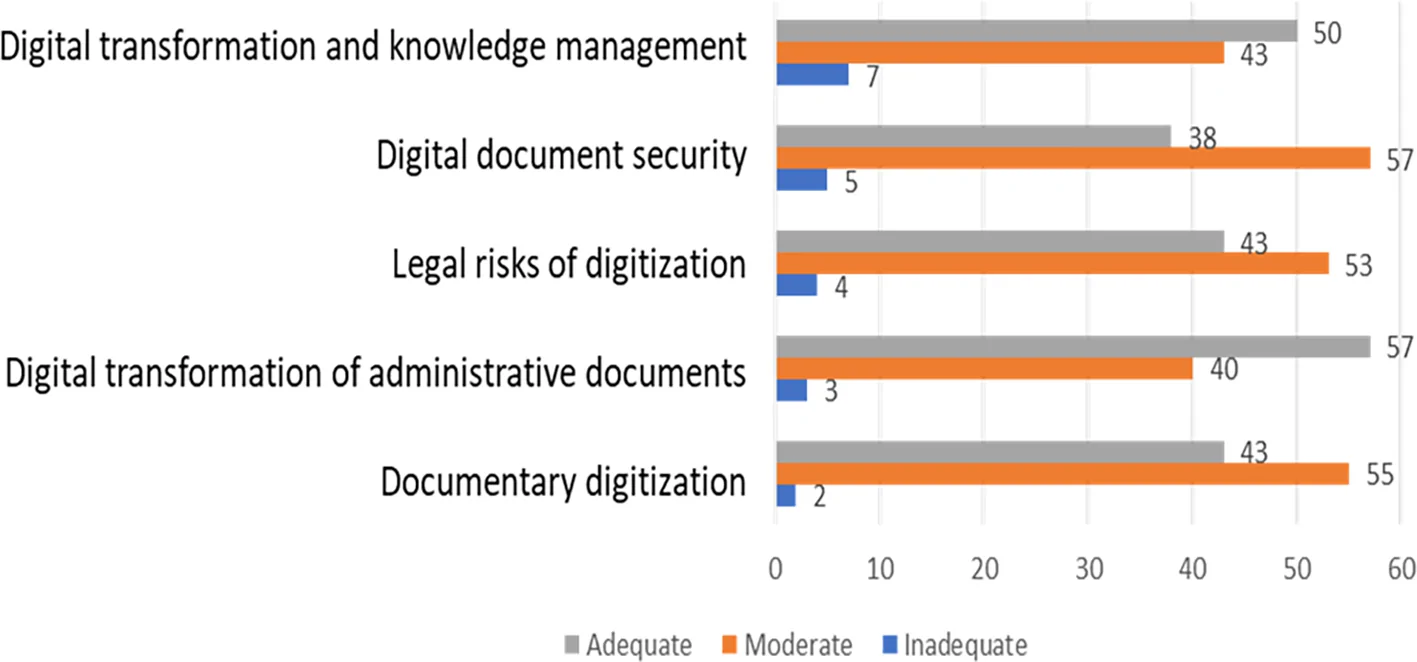
Level of use of document digitization in a municipality of Amazonas, Peru. Note: *Level of use of document digitization in a municipality of Amazonas, Peru.*

Figure 4 presents the reduction in expenditure levels observed by the municipality after the implementation of the digitisation of documents; there was a clear reduction in operating costs, which confirms the impact of digitisation on cost management.

**Figure 4.**
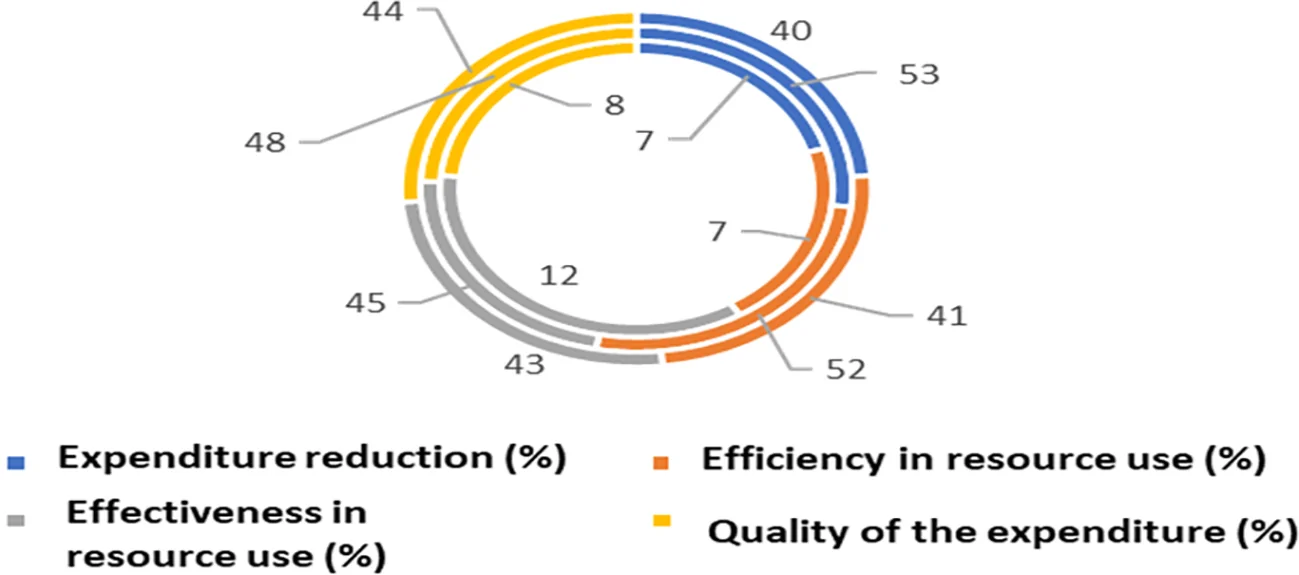
Level of expenditure reduction in a municipality of Amazonas, Peru. Note: *Level of expenditure reduction in a municipality of Amazonas, Peru.*

### Statistical analysis

**Model Summary: Influence of document digitization on cost reduction**

Table 1, illustrating the regression model indicated a significant influence of document digitization on cost reduction, with an R-squared value of 0.666; this suggests that a large proportion of the variance in cost reduction can be explained by the variables related to document digitization.

**Table 1.** Model summary: Influence of document digitization, legal risks, security, and knowledge management on cost reduction.

Model	R	R square	Adjusted R-squared	Standard error of the estimate	Statistics of change				
					Change in R-squared	Change in F	gl1	gl2	Sig. change in F
1	0,816 ^a^	0.666	0.651	0.360	0.666	44.863	4	90	0.000

Note: *Model summary: Influence of document digitization, legal risks, security, and knowledge management on cost reduction.*

Analysis of Variance (ANOVA): Significance of the Model on Cost Reduction

Table 2 illustrates the ANOVA results conform the significance of the regression model in explaining cost reduction, with a ´-value of 0.000; this indicates that the model significantly contributes to explaining the variation in cost reduction.

**Table 2.** Analysis of Variance (ANOVA): Significance of the model on cost reduction.

Model		Sum of squares	gl	Root mean square	F	Sig.
1	Regression	23.232	4	5.808	44.863	,000 ^b^
	Waste	11.652	90	0.129		
	Total	34.884	94			

Note: *Analysis of Variance (ANOVA): Significance of the model on cost reduction.*

Regression Coefficients: Influence od Digitization on Cost Reduction

Table 3 illustrates the regression coefficients show that the digital transformation of administrative documents and the associated processes significantly influence cost reduction; specifically, Legal Risks of Digitization (β = 0.395, p < 0.000) and Digital Transformation and Knowledge Management (β = 0.283, p < 0.004) had the most substantial positive impacts on cost reduction.

**Table 3.** Regression coefficients: Influence of document digitization and associated processes on cost reduction.

Model		Unstandardized coefficients		Standardized coefficients	t	Sig.
		B	Error	Beta		
1	(Constant)	0.047	0.199		0.234	0.815
	Digital transformation	0.050	0.075	0.046	0.669	0.505
	Legal risks	0.422	0.110	0.395	3.830	0.000
	Security	0.207	0.114	0.195	1.807	0.074
	Knowledge Management	0.274	0.093	0.283	2.947	0.004

Note: *Regression coefficients: Influence of document digitization and associated processes on cost reduction.*

Table 4, illustrating the analysis of document digitization´s influence on cost reduction shows a strong relationship (R = 0.758. R-squared = 0.575), indicating that document digitization is a significant factor in reducing municipal costs.

**Table 4.** Summary of the model: influence of document digitization on cost reduction.

Model	R	R square	Adjusted R-squared	Standard error of the estimate
1	,758 ^a^	0.575	0.571	0.399

Note: *Summary of the model: influence of document digitization on cost reduction.*

## Discussion and conclusion

The purpose of this study was to examine how document digitization influences cost reduction in a municipality located in Amazonas, Peru; Grounded in theories of digital management (Carr and Clayton) and organizational efficiency (Max Weber), this research provides empirical evidence on the extent to which digitizing administrative documents can streamline processes, lower operating costs and enhance overall public sector performance; a quantitative approach and a non-experimental cross-sectional design were adopted to evaluate the direct impact of digitization on cost reduction, and the findings strongly validate the study hypotheses.

The regression model demonstrated a high coefficient of determination (R = 0.758, R ^2^ = 0.575), indicating that document digitization explains over half of the variance in cost reduction; ANOVA results reinforced the statistical significance of this relationship (F = 125.987, p = 0.000); Moreover, regression coefficients showed a robust and positive influence of digitization on lowering municipal operating costs (β = 0.758, r = 11.224, p = 0.000); These results underscore the practical benefits of digitization, such as reduced physical storage needs, streamlined retrieval of records, and decreased expendityre on manual processes.

From a theoretical standpoint, these outcomes corroborate digital management theory ( Haug et al., 2024; Fazil et al., 2024; Mariani and Bianchi, 2023). Which posits that process automation and technology adoption drive operational efficiency. They also align with organizational efficiency theory ( Al Yami et al., 2022; Garengo & Sardi, 2021; Rahmati & Jalilvand, 2024), underscoring how standardized procedures and well-defined structures reduce waste and improve planning.

### Alignment with previous studies

Mohale (2024), Vicente et al. (2022) and Viale & Zouari (2020) likewise identified considerable benefits of digitization in operational efficiency and user satisfaction.

Huamán and Medina (2022) and Patrucco et al. (2021) highlighted significant cost savings when governance is optimized through digital technologies.

Wen et al. (2022) and Ramírez-Asís et al. (2022) demonstrated that digital transformation, combined with regulatory measures, increases productivity but requires coordination to avoid negative interactions.

Castro-Nagatomy et al. (2022), Kuhlmann and Bogumil (2021) and Sestino et al. (2023) both underscore the importance of effective change management and robust IT infrastructure in maximizing the returns of digitization initiatives.

Zainuddin et al. (2021), Ponti et al. (2022), Sternad et al. (2023) and Torok (2024) show that digitization not only reduces operational cost but also promotes transparency and accountability in reduces operational cost but also promotes transparency and accountability in local governance, an essential aspect corroborated by the present findings.

### Practical implications

1. **Security and legal compliance**
  Despite evident cost savings, moderate levels of digital security and legal risk management persist; Municipalities should prioritize advanced solutions such as Public Key Infrastructure (PKI) or blockchain to safeguard document integrity and authenticity.
2. **Infrastructure investment**
  Strengthening ICT infrastructure (hardware, software, and networking) is paramount for scaling up digitization efforts; this investment ensures smoother document management and higher resilience against system downtimes.
3. **Staff training and development**
  Ongoing ICT training programs would maximize the positive impact of digitization on administrative efficiency; equipping municipal employees with the necessary digital skills accelerates technology adoption and minimizes operational errors.
4. **Knowledge management systems**
  Integrating platforms for efficient data capture, storage, and retrieval fosters better decision-making processes. These further drives cost savings and boosts public confidence in local government operations.
5. **E-Government and citizen engagement**
  Beyond internal benefits, digitization initiatives that promote transparency, citizen participation, and ease of access to municipal services can enhance public trust and accountability.

### Limitations of the study

**Geographical scope:** The analysis focused on a single municipality in Amazonas, Peru. Results might not fully represent the reality in other Peruvian regions or different national contexts with varying technological and administrative capacities.

**Cross-sectional design:** Data was collected at a single point in time, limiting insights into long-term causal relationships.

**Implementation variability:** Differences in how departments adopt and manage digital systems may affect the consistency of digitization outcomes, potentially impacting the overall cost-saving estimates.

### Future research directions

1. **Longitudinal studies:** Investigate the enduring impact of document digitization on operational efficiency over time, capturing how digital transformation evolves and matures in municipalities.
2. **Comparative analyses:** Examine multiple municipalities or regions, both rural and urban, to identify contextual factors (e.g., ICT investment levels, policy frameworks) that influence digitization success.
3. **Risk and security focus:** Develop frameworks that thoroughly assess legal and cybersecurity risks, proposing mitigating strategies that ensure robust and resilient e-government initiatives.
4. **Citizen-centric outcomes:** Explore the relationship between digitization efforts and public satisfaction, trust and engagement, aiming to correlate financial metrics (cost reduction) with broader societal benefits (transparency participation).

## Conclusion

This study confirms that document digitisation is a powerful driver of cost reduction in public administration contexts; by streamlining record keeping, reducing reliance on paper and improving data accessibility, digitisation significantly increases organisational efficiency and resource management; while the results are in line with existing studies, important gaps remain, notably in the areas of security, legal risk management and consistent adoption; addressing these challenges through strategic investments, staff development and advanced technological solutions can further improve the financial and social returns of digitisation efforts¸ in this way, municipalities in Amazonas and similar regions can accelerate their transition to more transparent, efficient and accountable governance.

## Ethics and consent

Ethical approval for the project “Public Documents Digitization: Towards Efficiency and Reduction of Municipal Expenses” was granted by the Comité de Ética en Investigación de Contabilidad – Pregrado, Universidad César Vallejo (UCV), Chiclayo, Perú. The committee verified the protocol, instruments, informed-consent procedures, and authorization to conduct the study in its Reporte de Verificación de Requisitos dated 19 July 2024.

Institutional approval of the research project was subsequently issued by UCV through Resolution No. 051-2024-UCV-VA-CON-F02/CEP (Chiclayo, 26 July 2024), which serves as the formal ethics approval/reference number for this study.

UCV does not assign a separate standalone IRB/ethics code beyond the committee’s verification and the university resolution; therefore, no additional approval ID is available. Written informed consent was obtained from all participants prior to data collection; all respondents were ≥18 years old. The study adhered to the Declaration of Helsinki and to applicable national and institutional research ethics standards. No independent external review board participated; ethics oversight was provided internally by UCV’s committee.

## Additional materials and data

The additional data used in this study, including the complete questionnaire, informed consent form, instrument application guide, and supplementary tables of statistical results, are publicly available in the same data repository: https://doi.org/10.23728/B2SHARE.C38034D43FD548B5810400EB7B3D5E4B ( Condor, M. M. C. 2025).

## Process identifier

These materials allow for open replication and review of the study. The complete dataset is accessible without restrictions or embargoes under a CC-BY 4.0 licence.

## Data Availability

All data supporting the results of this study, including the values underlying the means, standard deviations, and other reported measures; the values used to generate the figures; and the data points extracted from the images for analysis, are available in Segundo Cajusol’s data availability repository. **Excel database** **B2SHARE EUDAT: DOI**: https://doi.org/10.23728/B2SHARE.C38034D43FD548B5810400EB7B3D5E4B ( Condor, M. M. C. 2025).
