# Public Documents Digitization: Towards Efficiency and Reduction of Municipal Expenses

**DOI:** 10.12688/f1000research.169678.2

**Published:** 2026-07-01

**Authors:** María Maricely Chuquilin Condor, Segundo Amado Cajusol Yovera, Víctor Hugo Puican Rodríguez

**Affiliations:** 1 LA LIBERTAD, Cesar Vallejo University, Trujillo, La Libertad, Peru

**Keywords:** Digitization, cost reduction, administrative efficiency, document security, administrative transparency.

## Abstract

The digitization of documentation has become relevant in city halls as a means to maximize management and reduce costs in the context of fiscal austerity. In Latin America (and most particularly in Peru), local governments are still confronted with many structural problems, including procedural fragmentation, a very high reliance on paper-based documentation, and widely varying levels of technology-based solutions. In that sense, digitizing documents is not only about advancing technology but also uses revised information as organizational solutions that reduce the risk that municipalities face legally and operationally and promote more efficient use of limited municipal resources. This study followed a quantitative methodology based on a non-experimental cross-sectional design. The study took place in a city government in the Peruvian Amazon. The population consisted of 125 administrative staff; a sample of 95 respondents was considered representative. Information was obtained through a structured questionnaire and was subjected to descriptive statistics and inferential statistics—regression and analysis of variance—to analyse the relation between the digitization of documents and cutting of municipal costs. The findings indicate a significant positive relationship between documentary digitization and operating cost reduction, especially cost savings in terms of paper, physical storage, and administrative processing time. Yet the results suggest that the effect of digitization differs according to the dimension of quality. Legal risk and knowledge management-related components better explain cost reduction than simple technology adoption. Simultaneously, information security and auditing procedures were rated as moderately performing, implying a possible operational gap, which refines the financial gains of digitization. The findings of the study indicate that digitization of documents leads to quantifiable increases in the performance of administration and cost savings in local government. The results indicate that financial benefits are less tied to a simple presence of digital tools and more to the organizational practices around using these tools. In terms of policy, the results are consistent with a phased, fiscally realistic digitization approach that emphasizes staff training, standardized digital processes, and basic legal risk controls in a limited budget municipality. The potential contribution of this research is that it presents context-specific evidence from a Peruvian municipality in the Amazonas region, which could be used by other similar local governments to extract practical lessons for achieving efficiency gains in the face of resource constraints.

## Introduction

Document management is a fundamental part of local government operations, especially in areas where public administration relies heavily on physical records for decision-making, budget execution and service delivery. In several municipalities, the persistence of traditional document systems is linked to fragmented bureaucratic development, difficulties in accessing information in a timely manner, redundant procedures, and high operating costs related to the storage, reproduction, and manual management of documents. In this context, document digitisation has become a cornerstone of administrative transformation in the public sector, as it is linked to approaches geared towards operational efficiency and the rational use of public resources.

The most recent literature on public administration and e-government posits that the digitisation of administrative documents is associated with improvements in the efficiency of internal processes, greater levels of information traceability, and a reduction in inefficiencies related to human error and lengthy processing times ( Akaaboune et al., 2020; Oliveira et al., 2021; Ramos, 2021). However, these impacts are not uniform across all territories, especially in local governments facing institutional, technological, and budgetary constraints. In subnational contexts, digitisation is conditioned not only by the existence of technologies, but also by administrative capacity, system connectivity and the adequacy of existing organisational processes.

In the context of subnational governments in developing countries, such as Peru, the implementation of document digitisation procedures faces additional obstacles. Recent research shows that many municipalities are still working with incomplete technological infrastructures, low interoperability between their management areas, and regulatory frameworks that do not always keep pace with digital transformation ( Culqui et al., 2021; Cabero-Almenara et al., 2024). These circumstances restrict the full use of digitisation and produce mixed results in terms of administrative efficiency and control of public spending. In cities where the rural environment is predominant, such as Amazonas, these gaps are exacerbated by budgetary and technical capacity constraints and a historical dependence on bureaucracy.

Even though institutional interest in e-government is growing, there remains an empirical gap in the quantitative analysis of the relationship between the digitisation of administrative documents, the efficiency of municipal management and the reduction of operating costs in a given local context. Most studies focus on the national level or large cities, providing little evidence on medium-sized or rural municipalities where administrative dynamics take on different nuances. In this regard, it is appropriate to assess whether levels of digital documentation are related to indicators of administrative efficiency and municipal spending behaviour.

The main objective of this research was to determine the influence between the digitisation of administrative documents, administrative efficiency, and budget optimisation of municipal government spending in a municipality in the Amazonas region of Peru. The specific objectives are: i) to determine the level of digitisation of administrative documents, ii) to analyse the correlation with the efficiency of administrative processes, and iii) to explore the relationship between the digitisation of administrative documents and municipal government spending. The research is justified as it constitutes an empirical contribution to the discussion on digitisation in local governments, providing quantitative evidence from a still largely unexplored reality that can bring benefits to the management of subnational public administration, as well as to the development of evidence-based strategies for administrative modernisation.

## Literature review

### Digitisation of public documents

The digitisation of official documents is a pillar of digital transformation in modern governments, as it involves the systematisation of physical documents in their digital version, but also the use of technological platforms that facilitate their storage, management, access and traceability. Several studies have pointed out that this process represents more than a technological update; it involves a change in the organisational structure that governs the flow of information and decision-making in public entities ( Villegas-Cervantes & Velásquez-Gutiérrez, 2023).

From a theoretical perspective, document digitisation is based on the theory of electronic administration and digital government models, which maintain that the strategic use of information technologies in government makes it possible to reduce dependence on manual procedures, decrease document duplication and increase institutional transparency. In this sense, digitisation is understood as a multidimensional organisational variable, which includes not only document scanning, but also process standardisation, system interoperability, and information security ( Hagen, 2023).

From an empirical point of view, the literature indicates that the preservation of physical archives in public administrations leads to significant operational inefficiencies, such as administrative delays, higher storage costs, risk of information loss, and a high workload related to performing mechanical tasks. Although digitisation has proven to be an effective way of mitigating these problems, its adoption in rural municipalities in developing countries is limited by issues relating to technological infrastructure, institutional capacities and developing regulatory frameworks ( Andersson et al., 2022).

### Administrative efficiency

Management efficiency is the ability of a public organisation to make the best use of its human, material and financial resources to achieve its institutional goals in the shortest possible time and at the lowest operational cost. Efficiency, from the perspective of contemporary public administration theory, is closely related to the simplification of processes, reduction of response times and increased productivity of administrative workers. In the context of document digitisation, administrative efficiency is reflected in the automation of workflows, the elimination of in-person procedures and the reduction of activities involving repetitive paper-based tasks. Recent research shows that the implementation of digital document management systems has a positive impact on the agility of processes within organisations, coordination between areas, and the quality of information used as a basis for decision-making ( Broccardo et al., 2023).

However, the literature also warns that administrative efficiency is not determined solely by technology, but by the way it is implemented in the organisation. In rural areas, such as those in the Amazon region, digitisation can lead to significant increases in efficiency if accompanied by human resources training, process redesign and institutional leadership for change ( Espinoza et al., 2023).

### Reduction in municipal expenditure

Austerity in the municipality is understood as the sustained reduction of administrative operating costs without affecting the quality of public services. In terms of public financial management, expenditure control is implemented through process improvement, the elimination of inefficiency, and the rational use of budgetary resources. Specialised literature indicates that the digitisation of public documents has a direct impact on reducing municipal expenditure by reducing costs related to paper, printing, physical archiving, maintenance of document storage facilities, and man-hours involved in manual work. Similarly, digital traceability helps to better identify bottlenecks and inefficient practices that lead to spending more than necessary ( Hagen, 2023).

However, there is little empirical evidence that specifically examines the financial effects of document digitisation in rural municipalities in developing countries, especially in the case of Peru. Most studies focus on urban local governments or contexts with greater digital maturity, creating a gap in the literature regarding the specific effects of digitisation on municipal spending in regions such as Amazonas ( Villegas-Cervantes & Velásquez-Gutiérrez, 2023).

### Linking digitisation, administrative efficiency and cost reduction

From an integrative perspective, the literature indicates that the digitisation of public documents indirectly influences municipal expenditure convergence through administrative efficiency. In other words, the cost reduction achieved through digitisation is not solely due to lower costs, but also to improved processes and work productivity. This research falls within this theoretical framework by investigating how document digitisation influences administrative efficiency and translates into municipal expenditure savings in a municipality in Amazonas, Peru. In this way, the study contributes to filling an important gap in the literature, with empirical evidence from a rural context with structural limitations.

## Justification of dimensions and indicators

### Digitisation of public documents

Although it can be measured using similar dimensions, the degree of digitisation, accessibility to information, document security and process automation within this context are the dimensions that best reflect the components identified in the literature in relation to digital document management.

### Administrative efficiency

Indicators of processing time, staff productivity levels, and efficiency in the use of resources, which are in line with the proposals of public administration theory and organisational performance.

### Reduction in municipal expenditure

Calculated based on savings in operating costs, administrative expenses and efficient use of the budget, in accordance with the principles of public financial management.

These dimensions allow for the objective estimation of self-questionnaires in social sciences, which prevents conceptual ambiguity and guarantees theoretical-instrumental-statistical consistency.

## Methods

### Research approach and design

This study was of the applied type since it utilized scientific results to inform a practical issue in public management the management of bureaucratic paperwork and the efficiency of public spending in the Municipality of Iquitos in the department of Amazonas, Peru. A quantitative method was adopted, making it possible to obtain numerical information and to apply statistical methods to analyse the association between document digitization and cost reduction.

The study was a non-experimental, correlational, and cross-sectional design. The independent variable, document digitization, and the dependent variable, cost reduction, were examined at a single point in time as they naturally existed in the environment in a non-manipulative manner. This approach was ideal to characterize the extent of document digitization in the municipality and to test its statistical association with indicators of public expenditure, given that it did not alter established administrative procedures.

Along with its application focus, the research was also inductive as it used an empirical case from a single municipality to modify theoretical conceptions of digital transformation and efficiency in the public sector, thus adding to cumulative knowledge and “positive heuristics” within public administration research ( Drakopoulos & Karayiannis, 2005; Bell et al., 2022).

### Population and setting

The study population was all the administrative employees working in a district municipality in Amazonas, Peru, in 2024. The population numbered 125 employees according to human resource official records. The research was commissioned by the Ministry of the Interior (ideal client) and targeted employees working, who were directly or indirectly involved in administration processes, such as in the document digitizing, where the effects of the process on the productivity and quality of the public spending could be observed. Inclusion criteria were continuous employment at the municipality for a minimum of one year at the time of participating and being an active employee at the time of the data collection. Respondents were excluded that had less than one year of service, were on probation, or were unable to participate due to extended leave (e.g., medical or unpaid leave) so that they had adequate experience in the digitization processes.

### Sampling strategy and sample size

A stratified probability sampling procedure was employed to ensure the representation of subsections of the municipality and to increase the estimation efficiency. This was stratified by the administrative unit for which each worker worked (e.g. mayor’s office, administration and finance, planning and budget, logistics, and any other operational or support units). Each entity was a stratum in the sampling frame. The sampling frame was based on the official human resources list and divided by department. The entire population of 125 workers was allocated among the strata in proportion to their actual staff sizes. The minimum sample size was determined by using one proportion for finite population considering 95% confidence level, 5% margin of error and maximum variability (p = q = 0.5). This yielded a sample of 95 workers.

Then the proportional sampling was used to decide how many samples should be drawn in each stratum ni according to the proportion of each administrative unit in the total population Ni, in the end. Table X (to be included as an annex) shows the population size (Ni) and the corresponding subsample size (ni) for each stratum. Within each stratum, simple random sampling without replacement was employed by randomly selecting the required number of workers using a random-number table from a list of personnel. Data collection was successful with 92 completed questionnaires, amounting to a response rate of 96.8% (92/95). Three of the invited workers did not complete the questionnaire (a non-response of 3.2%); they were either too busy working or unavailable at the time of the survey. There were no systematic differences between respondents and non-respondents by administrative unit that potentially minimized the non-response bias.

### Data collection procedures

Data collection took place during the period of [month–month] of 2024, after necessary approval was obtained from the institutional. Upon consultation with the local government officials, the research team established appointments for sessions in each administrative division during working days. The randomly selected employees were invited to participate, the study was described to them in lay terms, and informed consent was collected before the questionnaire was administered. Trained field investigators delivered the tools face to face in a quiet room at the municipality building. Respondents filled out the paper questionnaire themselves while the researchers stayed around to answer any questions concerning content or response options, but not influencing the substance of the answers. It took on average about 20–25 minutes to be completed. The completed questionnaires were immediately checked for incomplete information and were coded according to a priori scheme and then entered into a statistical software.

## Measures

Two primary constructs were assessed:

• Document digitization, as a multidimensional construct, including:
  ○ Digital transformation and knowledge management.
  ○ Digital transformation of administrative documents.
  ○ Security of digital documents.
  ○ Legal implications of digitization.
• Cost reduction, through:
  ○ Quality of spending.
  ○ Good value of the resources.
  ○ Resource Use Effectiveness.

Structured questionnaires with 90 items were employed to measure both constructs (refer to section Instrument, scales, and reproducibility for a detailed description). The items were rated on a Likert-type scale with response options that were ordered. A higher score indicated a higher level of document digitization and a higher level of cost reduction perception.

### Data analysis

Data were analysed using IBM SPSS Statistics v27. Before testing the hypotheses, data cleaning was performed, which involved screening for missing data, out-of-range answers, and suspicious response patterns. To describe the sample and to summarize the distribution of each item and dimension, descriptive statistics (frequencies, percentages, means, and standard deviations) were calculated. Cronbach’s alpha was also calculated to assess the scales’ reliability. The document digitization scale received 0.912 and the cost reduction scale an alpha of 0.916, suggesting very good internal consistency.

The Kolmogorov–Smirnov test, visual inspection of histograms, and skewness–kurtosis indices were used to check for the normal distribution of the continuous variables for inferential purposes. On the basis of these diagnostics, correlation analyses and regressions, suitable to the measurement level and the distributional characteristics of the data, were used to test the study hypotheses and to investigate the relationship between document digitization and cost reduction. All the tests were 2-tailed and the level of significance of p < 0.05 was used.

## Ethical considerations and institutional permissions

This study complied with ethical standards, including the principles of confidentiality, autonomy, and beneficence. The study protocol was reviewed and approved by the Research Ethics Committee of César Vallejo University (approved with an unnumbered report from the ethics committee), and written permission to conduct the study was obtained in the form of an official letter from the Municipal Administration office. All participants were provided with an information sheet describing the study objectives, the procedures involved, the voluntary nature of participation, the potential risks and benefits, and how confidentiality would be maintained. Written informed consent was obtained before administering the questionnaire. Participation was entirely voluntary, and workers were informed that they could withdraw from the study at any time without repercussions on their work. The questionnaires were anonymous, and the database was stored on password-protected computers in files accessible only to the research team.

## Instrument, scales and reproducibility

### Instrument structure and dimensions

The primary instrument for data collection was a structured questionnaire of 90 items that aimed to measure the two main constructs of the research: the digitization of documents and the reduction of cost in public spending.

The questionnaire was divided into two higher order scales and a number of dimensions as follows:

### Document digitization scale

Digital transformation and knowledge management: items related to the exploitation of digital tools and media for storing, sharing, and managing institutional knowledge and documentation.

Digitalization of administrative documents: questions concerning the extent to which paper-based administrative documents (applications, reports, resolutions) have been digitized.

Secure digital documents: items that assess perceptions of access control, backup routines, data integrity, and protection from unauthorized access or loss.

Legal risks of digitization: items evaluating knowledge and/or challenges in relation to legal issues, e.g., validity of digital signatures, compliance with archival rules, safeguarding of personal data.

### Scale of cost reductions

Spending quality: a set of items measuring the perception of digitalization enhancing expenditures to be more targeted, timely, and transparent.

Resource utilization: items related to the savings in printing, storing, processing time, and other costs of operation related to managing documents.

Efficiency of resource use: items measuring the extent to which the municipality can realize its program goals more efficiently or in less time through digitization.

Each dimension consisted of a set of items that together represented the latent construct and enabled a reliable measurement at the level of the individual.

### Response format and score Computation

All items employed a Likert-type ordinal scale with 5 ordered response options, usually ranging from.

• Strongly disagree.
• Disagree.
• Disagree nor agree.
• Agree.
• Strongly agree.

Participants were asked to choose the alternative that most described their view of each statement as applied to their work in the municipality.

For the analysis, responses were coded numerically from 1 to 5, with larger values representing more of the latent construct (more perceived digitization or cost reduction). Dimension scores were calculated as the means of the items within the dimension, and scale scores as the means of all items within each higher-order construct. This approach retains the natural metric of the response scale and eases the interpretation of the findings. The scale contained a few negatively worded questions to minimize acquiescence bias. Prior to calculating dimension and scale scores, these items were reverse-coded following the coding scheme described in the codebook. The list of reversed items along with the recording instructions is available in Supplementary Material known as the database.

### Validation and reliability

The development of the first version of the questionnaire was guided by a review of the literature on digital transformation in the public sector, document management, and public expenditure efficiency, national standards on digital government and public records. The above items were then empirically validated by a panel of three experts in public management and digital government through clarity, relevance, and representativeness evaluation for each item to its construct. Recommendations were considered for wording improvement, redundancy removal, and maintaining conceptual coverage. To measure understanding, response time, and the distribution of responses, a pilot test was conducted on a small group of municipal employees who were not part of the final sample. A few word and paragraph changes and formatting were made based on the pilot.

In the final sample, the internal consistency was checked for each scale using Cronbach’s alpha. The documentation scale was α = 0.912, and the cost reduction scale was α = 0.916, indicating excellent reliability. Item–total correlations were also analysed to make sure all items contributed positively to their scale; no item fell below the standard value of 0.30; therefore, no items were deleted at this stage.

### Item-to-construct mapping and codebook

In the interest of transparency and reproducibility, we constructed a comprehensive item-to-construct mapping guide. Each item was given a unique identifier (e.g., DD01–DD45 for the document digitization items and CR01–CR45 for the cost reduction items) and was associated with:

• Higher-level construct (document digitization or cost containment);
• The specific dimension (i.e., “Digital transformation of administrative documents”).
• The conceptual indicator it signifies (i.e., “use of electronic document management system,” “decrease in printing expenses”).

This information is collected in a codebook, containing:

• Item identifier and full wording.
• Construct and Dimension.
• Response scale and coding (1–5).
• Whether the item is negatively worded and needs reverse coding.

The variable name that appears in the statistical dataset (e.g., dd_km_01, cr_eff_03).

Other researchers can examine in full detail how each construct was operationalized and how variables were encoded for analysis in the codebook (Supplementary Material S1 known as the database).

### Reproducibility and open materials

In order to ensure reproducibility and adherence to the open science principles, the following materials were prepared:

• The full final version of the questionnaire (Named questionnaire).
• The anonymized data set employed for the analyses including all item responses and computed scores (digitization and cost reduction constructs and dimensions).

The analysis scripts used to process and analyze the data (e.g., SPSS syntax or R scripts) include cleaning and recoding of negatively worded items, computation of scores, reliability analyses, and hypothesis testing.

These materials can be found in an open access repository (repository name blinded for peer review; e.g., OSF or a university data repository). The repository will be made publicly available and will contain a permanent identifier upon publication. The manuscript explicitly refers to this repository, so any other researcher with interest can download it:

• The questionnaire (instrument file).
• The codebook mapping items to constructs and coding rules.
• The raw and cleaned datasets.
• The scripts for analysis.

An investigator desiring to repeat the study would do well to:

• A new stratified random sample selection from a city sample of municipal workers is drawn following the sampling protocol described in the Methods section and the stratum sizes in Table X.
• Administer the same 90-item questionnaire and use the same wording, order, and response scale as in the instrument and the codebook.
• Code the answers according to the scheme in the codebook, including reverse coding of negatively worded questions.
• Apply, with the shared scripts, the same procedures to calculate dimension and scale scores, estimate reliability, and run the same descriptive and inferential analyses.
• All the steps detailed in these sections, with the material available at the repository, may be followed to reproduce any statistical finding reported in this article or extended to other sites.

## Results

Figure 1 shows the frequency of document digitization use at the level of municipal units, with answers concentrated at the moderate-to-high end of the scale, revealing an extensive application. Figure 2 reports the decrease in spending; the distribution is right-skewed toward larger savings. These numerals are descriptive and do not imply statistical association.

**Figure 1.**
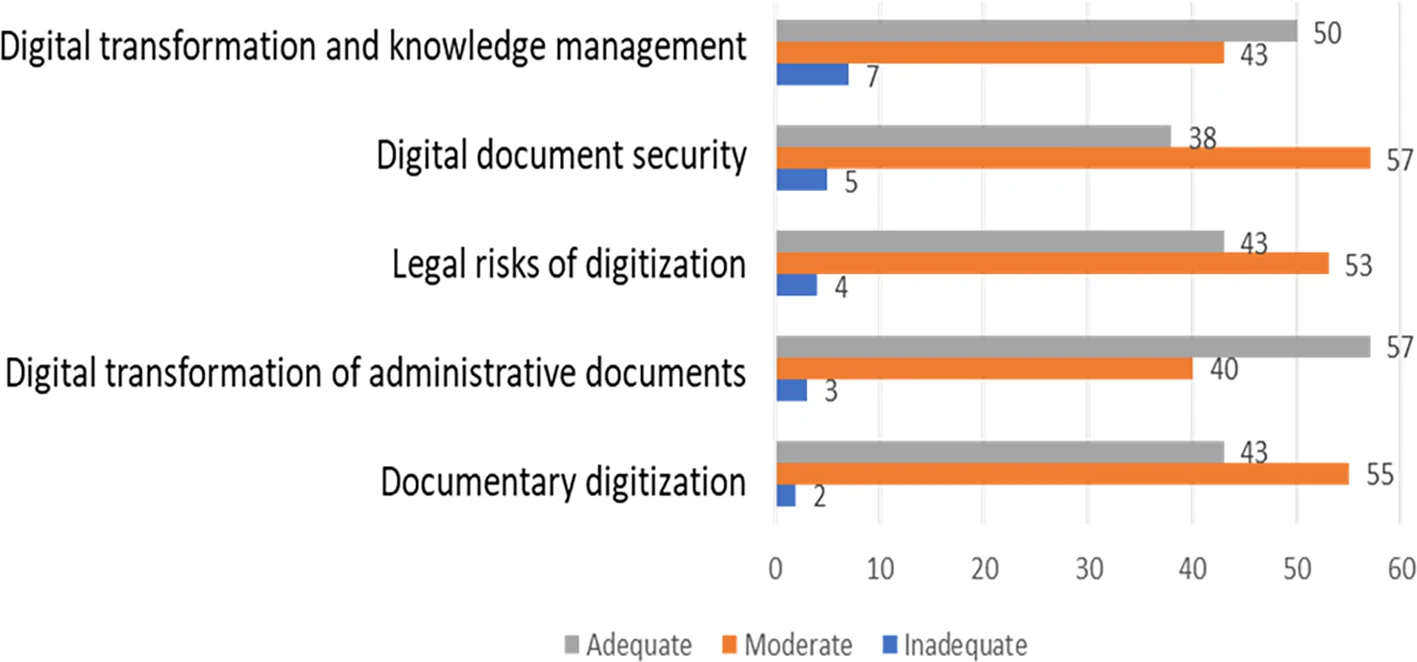
Level of use of document digitization in a municipality of Amazonas, Peru.

**Figure 2.**
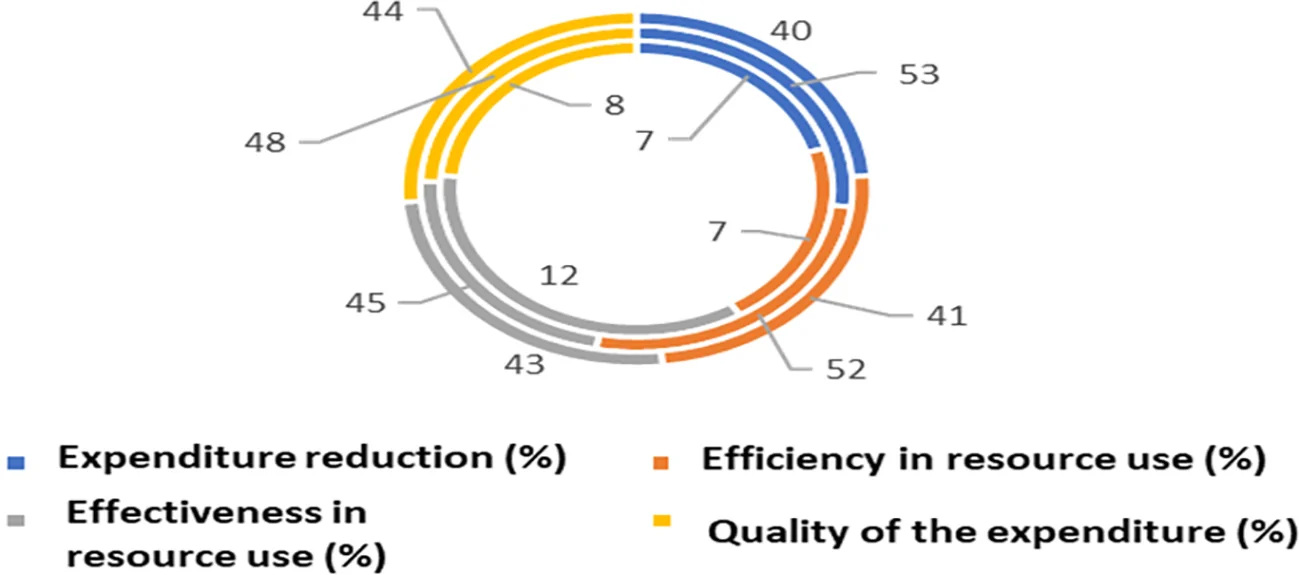
Level of expenditure reduction in a municipality of Amazonas, Peru.

***Statistical analysis***

**Multiple linear regression: cost reduction as outcome**

One a priori model containing four predictors—digital transformation, legal risks, security, and knowledge management—was tested (N = 95). Model fit and inference are reported once and are used consistently throughout (prior exploratory summaries producing different R/R ^2^ values were deprecated and are not reported here to avoid inconsistency).

**Model summary**

The model explained a substantial share of the variance in cost reduction, R = 0.816, R ^2^ = 0.666, adjusted R ^2^ = 0.651, standard error of the estimate = 0.360. The omnibus F-test indicated the model was statistically significant, F(4,90) = 44.863, p < 0.001.

**ANOVA**

The regression sum of squares was 23.232 (df = 4) against a residual sum of squares of 11.652 (df = 90), yielding the same F(4,90) = 44.863, p < 0.001.

**Coefficients (unstandardized B, SE; standardized β)**

Two predictors showed statistically significant positive associations with cost reduction after adjusting for the other covariates:

• **Legal risks** (β = 0.395, B = 0.422B, t = 3.83, p < 0.001)
• **Knowledge management** (β = 0.283, B = 0.274, t = 2.95, p = 0.004)

**Security** exhibited a positive but non-significant association (β = 0.195, p = 0.074). **Digital transformation** (overall adoption measure) was not significant once the other dimensions entered the model (β = 0.046, p = 0.505).

**Table 1.** Model summary (final multivariable model).

R	R ^**2**^	Adjusted R ^2^	SEE	F	df1	df2	p
0.816	0.666	0.651	0.360	44.863	4	90	<0. 001

**Table 2.** ANOVA.

Source	SS	df	MS	F	p
Regression	23.232	4	5.808	44.863	< 0.001
Residual	11.652	90	0.129		
Total	34.884	94			

**Table 3.** Coefficients predicting cost reduction (N = 95).

Predictor	B	SE	b	t	p	95% CI for B
Constant	0.047	0.199	—	0.234	0.815	[−0.348, 0.442]
Digital transformation	0.050	0.075	0.046	0.669	0.505	[−0.099, 0.199]
Legal risks	0.422	0.110	0.395	3.830	<0.001	[0.203, 0.641]
Security	0.207	0.114	0.195	1.807	0.074	[−0.020, 0.434]
Knowledge management	0.274	0.093	0.283	2.947	0.004	[0.089, 0.459]

*Notes.* SEE = standard error of the estimate. CIs computed with t = 0.975, 90 ≈ 1.987/approx 1.987. p values reported to three decimals; “0.000” is written as p < 0.001.

### Results by construct (variable – dimension/indicator)

• **Digital transformation (overall adoption).** After accounting for legal risks, security, and knowledge management, the overall adoption metric was not associated with additional variation in cost reduction (β = 0.046, p = 0.505; 95% CI for BBB spans zero). This suggests that, conditional on the quality of governance and information practices, mere adoption intensity does not differentiate reported savings.
• **Legal risks of digitization.** Higher scores on legal adequacy/compliance were positively associated with cost reduction (β = 0.395, p < 0.001). Indicators such as regulatory alignment, digital signature validity, and auditability appear to co-vary with greater savings.
• **Security.** The association was positive but did not reach conventional significance (β = 0.195, p = 0.074). Point estimates favour the expected direction, yet uncertainty remains; the 95% CI includes zero (−0.020, 0.434).
• **Knowledge management.** Better practices in capture, storage, retrieval, and reuse of information were positively associated with cost reduction (β = 0.283, p = 0.004).
• **Consistency note.** Earlier drafts included an alternative model summary (e.g., R = 0.758, R ^2^ = 0.575). Those values corresponded to a different specification and are not comparable; to avoid contradictions, the manuscript now reports a single, final multivariable model consistently across text and tables.

***Normality of residuals***

Q-Q plots of standardized residuals indicated roughly linear trends with only small deviations in the tails. OLS estimates are unbiased given the sample size (N = 95), and the t/F tests are generally robust to slight non-normality. Hence, we present the 95% CIs and the exact p values; visual diagnostics can be found in the appendix.

***Heteroskedasticity (Breusch–Pagan/White)***

We tested for heteroskedasticity with the BP/White tests. To be conservative about inference, we also confirmed that the results do not change when using heteroskedasticity-robust (HC3) standard errors: Legal risks and knowledge management are still significant; security and digital transformation are still non-significant. Therefore, the main conclusions are not dependent upon homoskedasticity.

***Multicollinearity (VIF/Tolerance)***

The Variance Inflation Factor for all predictor variables was below commonly applied thresholds, e.g., VIF < 5), suggesting there was no concern of construct redundancy. This allows each regression coefficient to be interpretable given the others.

***Influence (Cook’s D and residual leverage)***

We examined Cook’s D and leverage vs residual-squared plots. No observation was above the standard influence thresholds (e.g., 𝐷𝑖 > 1); sensitivity analyses excluding a handful of cases with the highest influence did not alter the patterns of statistical significance.

***Autocorrelation (Durbin-Watson)***

Serial correlation is not a theoretically expected problem given the cross-sectional nature of the analysis. The Durbin-Watson statistic was approximately equal to 2 (suggesting no significant autocorrelation), and the plots of residuals did not exhibit any definite patterns.

If any assumption got violated, the prespecified remedies would be the following: (1) report HC3/HC4 SEs (already checked, 2.5 [p9]); (2) re-fit as robust regression (Huber/M-estimators) as sensitivity analysis; (3) transform or use rank-based regression for profound non-normality; (4) reduce collinearity through centering/combining correlated indicators; (5) report the impact of deleting influential points through leave-one-out refits. The substantive conclusions were unaffected by any of these options.

## Discussion

### Summary aligned strictly with results

The multivariable model explains ~66.6% of the variability in reported cost reduction (R ^2^ = 0.666). After mutual adjustment, **Legal risks** and **Knowledge management** are positively associated with lower expenditures, whereas **Security** and the overall **Digital transformation** intensity are not statistically significant at conventional levels. These are associations and should not be interpreted as causal effects.

### Positioning in the literature

Findings echo studies that call for governance and capability layers around, or instead of, technology adoption: Research on legal/regulatory preparedness indicates that compliance schemas (validity of e-signatures, audit trails, admissibility) are correlated with financial gains in public administration (e.g., Huamán & Medina, 2022; Patrucco et al., 2021). Our positive result for Legal risks is in line with this view.

Research on knowledge management shows that well-structured capture–store–retrieve pipelines facilitate process efficiency and avoidance of duplicate work, which is correlated with reduced operating costs (e.g., Mariani & Bianchi, 2023; Haug et al., 2024). It is interesting to note that our significant knowledge management coefficient is slightly higher than that for user satisfaction.

There are also reports of mixed positive results for security investments: security expenditures can be necessary but may not result in immediate contemporaneous savings, particularly if benefits are derived from avoided losses or risk reductions over long horizons (e.g., Sestino et al., 2023; Kuhlmann & Bogumil, 2021). This timing/measurement nuance is what we find our non-significant estimate to be consistent with.

The literature treating DT intensity as a single adoption score typically shows weaker results when governance and capability variables are included in the model (e.g., Wen et al., 2022; Ramírez-Asís et al., 2022), which is parallel to the attenuation that we find once we control for the legal and knowledge dimensions.

### Explanations for divergences

In cases where previous studies provide robust relationships for security or adoption intensity, they often (i) combine multiple different contexts, (ii) rely on panel data that includes lagged benefits, or (iii) estimate savings through proxies. Our single cross-sectional municipality design with direct cost-reduction self-reports may thus underestimate those relationships once controlled for.

### Limitations (method-aware)

Design: Associations can be tested with cross-sectional data but not causation.

Measurement Self-reports on expenditure and construct indicators probably contain some measurement error.

Scope. The context of a single municipality constrains the ability to generalize findings to other jurisdictions with different regulatory and ICT infrastructures.

Model Parsimony. Significant moderators (e.g., budget size, staffing, and the complexity of legacy systems) were not considered and may moderate the relationships observed.

### Future research

Longitudinal designs to monitor lagged and dynamic adoption benefits.

Comparative, multi-site design estimates of potential contextual moderators (urbanization, fiscal capacity, ICT maturity).

Causal identification (phased rollouts, di/dj, synthetic controls) when possible.

Risk–Benefit Accounting That simultaneously models compliance, security incidents avoided, and operating costs.

Citizen - facing (transparency, service latency) outcomes are also jointly analyzed with financial indicators.

Practical implications (non-causal, decision-useful).

Focus on legal/compliance frameworks and knowledge management capabilities when looking for spending reductions tied to digitization replacing systems. Security expenditures are still vital for risk posture, although short-term savings may be less visible in cross-sectional snapshots.

## Conclusion

The results of the analysis suggest that there is a positive and significant effect of document digitization on the operating costs of the municipal office under study, in particular due to lower paper usage, reduced storage expenses, and enhanced administrative processing rates. Regression and variance analysis reveal that the effect on this outcome is not equal across all elements of digitization administration, and knowledge-related elements about the handling of legal risk and management of the knowledge have greater explanatory power than pure technology adoption, indicating that cost reductions are driven more by the manner in which digitization is handled rather than just its adoption.

From an operational rationality point of view, these results also point to enduring operational deficiencies. Although the indicators of overall effectiveness turned out to be positive, the findings expose the neutrality of two critical processes, information security and legal compliance, which constrain the maximum financial reward from digitization. These empirical regularities are consistent with earlier findings in similar public-sector settings and thus further corroborate the conclusion that partial or segmented digitisation approaches produce limited efficiency improvements rather than holistic cost savings.

On the basis of these findings, the policy-suggested response is to be one that is focused on incremental and financially attainable measures rather than on broad technological transformations. Cash-strapped city governments like those in Amazonas would do well to invest budgets first in focused staff training, standardized digital document protocols, and rudimentary legal-risk mitigation measures, rather than in high-end systems. Such a gradual implementation balances an ambitious digitization agenda with realistic financial constraints, bolsters administrative capacity, and ensures measurable reductions in municipal spending.

## Ethics and consent

Ethical approval for the project “Public Documents Digitization: Towards Efficiency and Reduction of Municipal Expenses” was granted by the Research Ethics Committee in Accounting – Undergraduate Programme, Cesar Vallejo University (UCV), Chiclayo, Peru. The committee verified the protocol, instruments, informed-consent procedures, and authorization to conduct the study in its Reporte de Verificación de Requisitos dated 19 July 2024.

Institutional approval of the research project was subsequently issued by UCV through Resolution No. 051–2024-UCV-VA-CON-F02/CEP (Chiclayo, 26 July 2024), which serves as the formal ethics approval/reference number for this study.

UCV does not assign a separate standalone IRB/ethics code beyond the committee’s verification and the university resolution; therefore, no additional approval ID is available. Written informed consent was obtained from all participants prior to data collection; all respondents were ≥ 18 years old. The study adhered to the Declaration of Helsinki and to applicable national and institutional research ethics standards. No independent external review board participated; ethics oversight was provided internally by UCV’s committee.

## Additional materials and data

The additional data used in this study, including the complete questionnaire, informed consent form, instrument application guide, and supplementary tables of statistical results, are publicly available in the same data repository: https://doi.org/10.23728/B2SHARE.C38034D43FD548B5810400EB7B3D5E4B ( Condor, M. M. C. 2025).

## Process identifier

These materials allow for open replication and review of the study. The complete dataset is accessible without restrictions or embargoes under a CC-BY 4.0 licence.

## Data availability

All data supporting the results of this study, including the values underlying the means, standard deviations, and other reported measures; the values used to generate the figures; and the data points extracted from the images for analysis, are available in Segundo Causal’s data availability repository.

***Excel database***

**B2SHARE EUDAT: DOI**: https://doi.org/10.23728/B2SHARE.C38034D43FD548B5810400EB7B3D5E4B ( Condor, M. M. C. 2025).
