# Public Documents Digitization: Towards Efficiency and Reduction of Municipal Expenses

**DOI:** 10.12688/f1000research.169678.4

**Published:** 2026-11-04

**Authors:** María Maricely Chuquilin Condor, Segundo Amado Cajusol Yovera, Víctor Hugo Puican Rodríguez

**Affiliations:** 1 LA LIBERTAD, Cesar Vallejo University, Trujillo, La Libertad, Peru

**Keywords:** Digitization, cost reduction, administrative efficiency, document security, administrative transparency.

## Abstract

**Objective:**

To examine the association between four dimensions of document digitization and staff perceptions regarding resource use, goal achievement, and the quality of spending in a Peruvian municipality.

**Methods:**

A quantitative, cross-sectional, non-experimental study was conducted. All 125 administrative staff members were invited to participate; 95 completed an online questionnaire consisting of 90 items (response rate: 76.0%). A multiple linear regression model was estimated, and HC3 standard errors were used for inference regarding the coefficients.

**Results:**

The model had an R ^2^ of 0.763. After mutual adjustment, the dimensions of legal risks of digitization (p = 0.010) and digital transformation and knowledge management (p = 0.015) showed positive associations with the outcome. The digital transformation of administrative documents (p = 0.920) did not show an additional statistically significant association. The association with document security was positive but not conclusive (p = 0.059).

**Conclusion:**

The findings identify associations between dimensions of digitization and perceptions of resource management within a single institution. The cross-sectional design does not allow for the establishment of causality, and the questionnaire does not quantify actual savings. The assessment of potential cost reductions requires accounting records and administrative indicators measured over time.

## Introduction

Document management is a fundamental part of local government operations, especially in areas where public administration relies heavily on physical records for decision-making, budget execution and service delivery. In several municipalities, the persistence of traditional document systems is linked to fragmented bureaucratic development, difficulties in accessing information in a timely manner, redundant procedures, and high operating costs related to the storage, reproduction, and manual management of documents. In this context, document digitisation has become a cornerstone of administrative transformation in the public sector, as it is linked to approaches geared towards operational efficiency and the rational use of public resources.

The most recent literature on public administration and e-government posits that the digitisation of administrative documents is associated with improvements in the efficiency of internal processes, greater levels of information traceability, and a reduction in inefficiencies related to human error and lengthy processing times ( Androniceanu y Georgescu, 2022; Oliveira et al., 2021; Ramos, 2021). However, these impacts are not uniform across all territories, especially in local governments facing institutional, technological, and budgetary constraints. In subnational contexts, digitisation is conditioned not only by the existence of technologies, but also by administrative capacity, system connectivity and the adequacy of existing organisational processes.

In the context of subnational governments in developing countries, such as Peru, the implementation of document digitisation procedures faces additional obstacles. Recent research shows that many municipalities are still working with incomplete technological infrastructures, low interoperability between their management areas, and regulatory frameworks that do not always keep pace with digital transformation ( Culqui et al., 2021; Cabero-Almenara et al., 2024). These circumstances restrict the full use of digitisation and produce mixed results in terms of administrative efficiency and control of public spending. In local governments, budgetary and technical constraints can make it difficult to implement digital document management systems.

Even though institutional interest in e-government is growing, there remains an empirical gap in the quantitative analysis of the relationship between the digitisation of administrative documents, the efficiency of municipal management and the reduction of operating costs in a given local context. Most studies focus on the national level or large cities, providing little evidence on medium-sized or rural municipalities where administrative dynamics take on different nuances. In this regard, it is appropriate to assess whether levels of digital documentation are related to indicators of administrative efficiency and municipal spending behaviour.

The main objective was to examine the association between four dimensions of document digitization and workers’ perceptions of resource use, the achievement of objectives, and the quality of spending in a municipality in the province of Utcubamba, Amazonas Department, Peru. The specific objectives are: i) to determine the level of digitisation of administrative documents, ii) to analyse the correlation with the efficiency of administrative processes, and iii) to explore the relationship between the digitisation of administrative documents and municipal government spending. The research is justified as it constitutes an empirical contribution to the discussion on digitisation in local governments, providing quantitative evidence from a still largely unexplored reality that can bring benefits to the management of subnational public administration, as well as to the development of evidence-based strategies for administrative modernisation.

## Literature review

### Digitisation of public documents

The digitisation of official documents is a pillar of digital transformation in modern governments, as it involves the systematisation of physical documents in their digital version, but also the use of technological platforms that facilitate their storage, management, access and traceability. Several studies have pointed out that this process represents more than a technological update; it involves a change in the organisational structure that governs the flow of information and decision-making in public entities ( Villegas-Cervantes & Velásquez-Gutiérrez, 2023).

From a theoretical perspective, document digitisation is based on the theory of electronic administration and digital government models, which maintain that the strategic use of information technologies in government makes it possible to reduce dependence on manual procedures, decrease document duplication and increase institutional transparency. In this sense, digitisation is understood as a multidimensional organisational variable, which includes not only document scanning, but also process standardisation, system interoperability, and information security ( Hagen, 2023).

From an empirical point of view, the literature indicates that the preservation of physical archives in public administrations leads to significant operational inefficiencies, such as administrative delays, higher storage costs, risk of information loss, and a high workload related to performing mechanical tasks. Although digitisation has proven to be an effective way of mitigating these problems, its adoption in rural municipalities in developing countries is limited by issues relating to technological infrastructure, institutional capacities and developing regulatory frameworks ( Andersson et al., 2022).

### Administrative efficiency

Management efficiency is the ability of a public organisation to make the best use of its human, material and financial resources to achieve its institutional goals in the shortest possible time and at the lowest operational cost. Efficiency, from the perspective of contemporary public administration theory, is closely related to the simplification of processes, reduction of response times and increased productivity of administrative workers. In the context of document digitisation, administrative efficiency is reflected in the automation of workflows, the elimination of in-person procedures and the reduction of activities involving repetitive paper-based tasks. Recent research shows that the implementation of digital document management systems has a positive impact on the agility of processes within organisations, coordination between areas, and the quality of information used as a basis for decision-making ( Broccardo et al., 2023).

However, the literature also warns that administrative efficiency is not determined solely by technology, but by the way it is implemented in the organisation. In rural areas, such as those in the Amazon region, digitisation can lead to significant increases in efficiency if accompanied by human resources training, process redesign and institutional leadership for change ( Espinoza et al., 2023).

### Reduction in municipal expenditure

Austerity in the municipality is understood as the sustained reduction of administrative operating costs without affecting the quality of public services. In terms of public financial management, expenditure control is implemented through process improvement, the elimination of inefficiency, and the rational use of budgetary resources. Specialised literature indicates that the digitisation of public documents has a direct impact on reducing municipal expenditure by reducing costs related to paper, printing, physical archiving, maintenance of document storage facilities, and man-hours involved in manual work. Similarly, digital traceability helps to better identify bottlenecks and inefficient practices that lead to spending more than necessary ( Hagen, 2023).

However, there is little empirical evidence that specifically examines the financial effects of document digitisation in rural municipalities in developing countries, especially in the case of Peru. Most studies focus on urban local governments or contexts with greater digital maturity, creating a gap in the literature regarding the specific effects of digitisation on municipal spending in regions such as Amazonas ( Villegas-Cervantes & Velásquez-Gutiérrez, 2023).

### Linking digitisation, administrative efficiency and cost reduction

From an integrative perspective, the literature indicates that the digitisation of public documents indirectly influences municipal expenditure convergence through administrative efficiency. In other words, the cost reduction achieved through digitisation is not solely due to lower costs, but also to improved processes and work productivity. The purpose is to contribute to this theoretical framework by evaluating the relationship between the various aspects of document digitization and staff perceptions regarding the use of resources, the achievement of objectives, and the quality of spending in a municipality in the province of Utcubamba, Amazonas Department, Peru. In this way, the study contributes to filling an important gap in the literature, with empirical evidence from a rural context with structural limitations.

## Justification of dimensions and indicators

### Digitisation of public documents

Although it can be measured using similar dimensions, the degree of digitisation, accessibility to information, document security and process automation within this context are the dimensions that best reflect the components identified in the literature in relation to digital document management.

### Administrative efficiency

Indicators of processing time, staff productivity levels, and efficiency in the use of resources, which are in line with the proposals of public administration theory and organisational performance.

### Reduction in municipal expenditure

Calculated based on savings in operating costs, administrative expenses and efficient use of the budget, in accordance with the principles of public financial management.

These dimensions allow for the objective estimation of self-questionnaires in social sciences, which prevents conceptual ambiguity and guarantees theoretical-instrumental-statistical consistency.

## Methods

### Research approach and design

A quantitative, observational, correlational, and cross-sectional study was conducted involving administrative employees of a Peruvian municipality. Using a survey, the study analyzed the possible relationships between different dimensions of document digitization and staff perceptions regarding resource management and the quality of municipal spending. The variables were measured at a single point in time, without the researchers intervening in administrative processes. For this reason, the study design allows for the description of associations but does not permit the assertion of causal effects or the determination of actual monetary savings ( Drakopoulos & Karayiannis, 2005; Bell et al., 2022).

### Population and setting

The target population consisted of 125 administrative employees of a Peruvian city government in 2024, according to institutional records. The study was limited to the organization and the employees’ perceptions regarding document digitization and the management of municipal resources.

### Contextual scope of the study

The study was conducted in 2024 in a municipality in the province of Utcubamba, in the department of Amazonas (Peru). The organization’s 125 administrative employees were invited to participate, and 95 of them completed the questionnaire. Therefore, the population and sample correspond to the internal administrative structure of the municipality under study, but do not constitute a representative sample of all Peruvian municipalities. The participants were from a single municipality. The results may not be generalizable to other municipalities due to differences in infrastructure, resources, organization, and digital maturity among local governments. Similarly, the lack of comparative information on workers who did not respond makes it impossible to rule out nonresponse bias.

### Sampling strategy and sample size

A population of 125 administrative workers in a municipality was studied. All were invited to participate; no stratified random selection was performed. Ninety-five workers (76.0% of the population, 95/125) completed the questionnaire. The remaining 30 workers did not respond. The database used for the analysis consists of 95 records, each containing responses to all 90 items on the questionnaire.

### Data collection procedures

The questionnaire was completed online via a Google Form and contained 90 statements with five possible responses. A total of 95 workers responded. The responses were entered into a database for review and analysis. Neither the presence of interviewers nor the distribution of paper questionnaires was required for participation.

### Measures

Document digitization was assessed using 48 items, distributed across four dimensions: digitization of administrative documents at the TIDC (P1–P12), legal aspects of digitization (P13–P24), security of digital documents (P25–P36), and digitization and knowledge management (P37–P48). The results for this were measured using 42 items related to staff perceptions regarding the use of municipal resources (P49–P63), the achievement of goals and results with available resources (P64–P75), and the quality and transparency of spending (P76–P90). Each statement had five response options, coded from 1 (“strongly disagree”) to 5 (“strongly agree”). The dimension scores, as well as the overall score used in the analyses, were calculated as the means of their respective items.

### Data analysis

It was verified that all 95 records contained responses for each of the 90 items and that their values ranged from 1 to 5. Item means were calculated for each dimension, along with descriptive statistics. Internal reliability was estimated using Cronbach’s alpha. In the analysis sample, the alpha was 0.974 for document digitization (48 items) and 0.987 for the scale measuring perceptions of resource management and the quality of spending (42 items). These values are for the 95 participants and differ from those in the pilot study. A multiple linear regression analysis was conducted that simultaneously included the four dimensions of document digitization as predictors and the mean of items P49–P90 as the outcome variable. Residuals versus adjusted values, the Q-Q plot, the Breusch-Pagan and White test, variance inflation factors, and Cook’s D were evaluated. The main estimates for the coefficients are presented with HC3 standard errors and 95% confidence intervals. Sensitivity analyses with similar characteristics were performed using resampling of participants and a “leaving-one-out” method for suspects. The significance level for the tests was 0.05.

## Declarations

### Use of generative AI

The authors confirm that they have used ChatGPT (OpenAI, GPT-5.3 version) as a tool to refine the language throughout the manuscript in order to improve the clarity and academic rigour of the text. Generative AI tools were not used in the development of the study design, data collection, data analysis, interpretation of results, or formulation of scientific conclusions. The scientific content, methodology and results presented in the study are the work of the authors themselves; they guarantee that they are accurate, truthful and consistent with academic and ethical standards.

## Ethical considerations and institutional permissions

This study complied with ethical standards, including the principles of confidentiality, autonomy, and beneficence. The study protocol was reviewed and approved by the Research Ethics Committee of César Vallejo University (approved with an unnumbered report from the ethics committee), and written permission to conduct the study was obtained in the form of an official letter from the Municipal Administration office. All participants were provided with an information sheet describing the study objectives, the procedures involved, the voluntary nature of participation, the potential risks and benefits, and how confidentiality would be maintained. Written informed consent was obtained before administering the questionnaire. Participation was entirely voluntary, and workers were informed that they could withdraw from the study at any time without repercussions on their work. The questionnaires were anonymous, and the database was stored on password-protected computers in files accessible only to the research team.

## Instrument, scales and reproducibility

### Instrument structure and dimensions

A structured questionnaire consisting of 90 questions was sent to administrative staff. Questions P1–P48 pertain to document digitization and are divided into four dimensions: digital transformation of administrative documents (P1–P12), legal aspects of digitization (P13–P24), security of digital documents (P25–P36), and digital transformation and knowledge management (P37–P48). Questions P49–P90 capture perceptions regarding the use of resources and municipal spending: efficient use of resources (P49–P63), achievement of objectives and results with available resources (P64–P75), and quality and transparency in spending (P76–P90). These latter questions do not measure a monetary reduction in municipal expenditures.

### Response format and score computation

Each statement was rated using five ordered and coded options, ranging from 1 (“strongly disagree”) to 5 (“strongly agree”). The score for each dimension corresponded to the mean of the responses to its items, and the score for a group of dimensions corresponded to the mean of its 48 or 42 items, as applicable. The original response codes were retained for the analysis of the provided data and were not reverse-coded. A higher score indicates greater agreement with the statements on the corresponding scale and does not represent any amount of money saved.

### Validation and reliability

The instrument’s documentation consists of evaluation forms detailing the content and results of a pilot study conducted with ten participants. In that pilot study, Cronbach’s alpha for the 48 items related to document digitization was 0.903, and for the 42 items related to resource use and municipal spending, it was 0.972. When recalculated for the present analysis, which included 95 participants, the alpha coefficients were 0.974 and 0.987, respectively. The reports on the results of the pilot sample and the analytical sample are presented separately, as they represent two distinct sets of responses. These coefficients characterize internal consistency; on their own, they do not indicate the structure or validity of the constructs. The results from the pilot sample contain item-total correlations below 0.30, some of which are negative; therefore, it is not claimed that all items met this criterion.

### Item-to-construct mapping and codebook

The items are identified in the questionnaire and in the database with the codes P1–P90. Their assignment to dimensions, as well as the item intervals that will be used to calculate the scores, are specified in the section “Structure and Dimensions of the Instrument.” To reproduce the results: use the original response values 1–5 and calculate the averages within the intervals indicated in that document. The identifiers DD01–DD45 and CR01–CR45, which were present in a previous version of the manuscript, do not correspond to the structure of the instrument used and have been removed.

### Reproducibility and open materials

The questionnaire, the response dataset, the tables, figures, and documentation for evaluating the instrument are available in the B2SHARE public repository: https://doi.org/10.23728/b2share.w5xx7-krz29. The database contains individual responses identified as P1–P90 and the scores derived from each dimension. The formulation of the variables and the specifications for the analysis are described in this paper.

### Additional information

This section contains Figures 3 and 4, which have been added to this paper.

María Maricely Chuquilin Condor; Segundo Amado Cajusol Yovera; Víctor Hugo Puican Rodríguez. (2026). Additional Information [Dataset]. B2SHARE v2. https://doi.org/10.23728/b2share.9hqet-jpc79

## Results and discussion

### Results

Figure 1 shows the responses from administrative staff regarding the use of document digitization. Figure 2 shows their responses regarding resource management and the quality of municipal spending. Both figures summarize the survey data provided by the respondents themselves; they do not measure actual reductions in spending nor do they establish a relationship between the variables.

**Figure 1.**
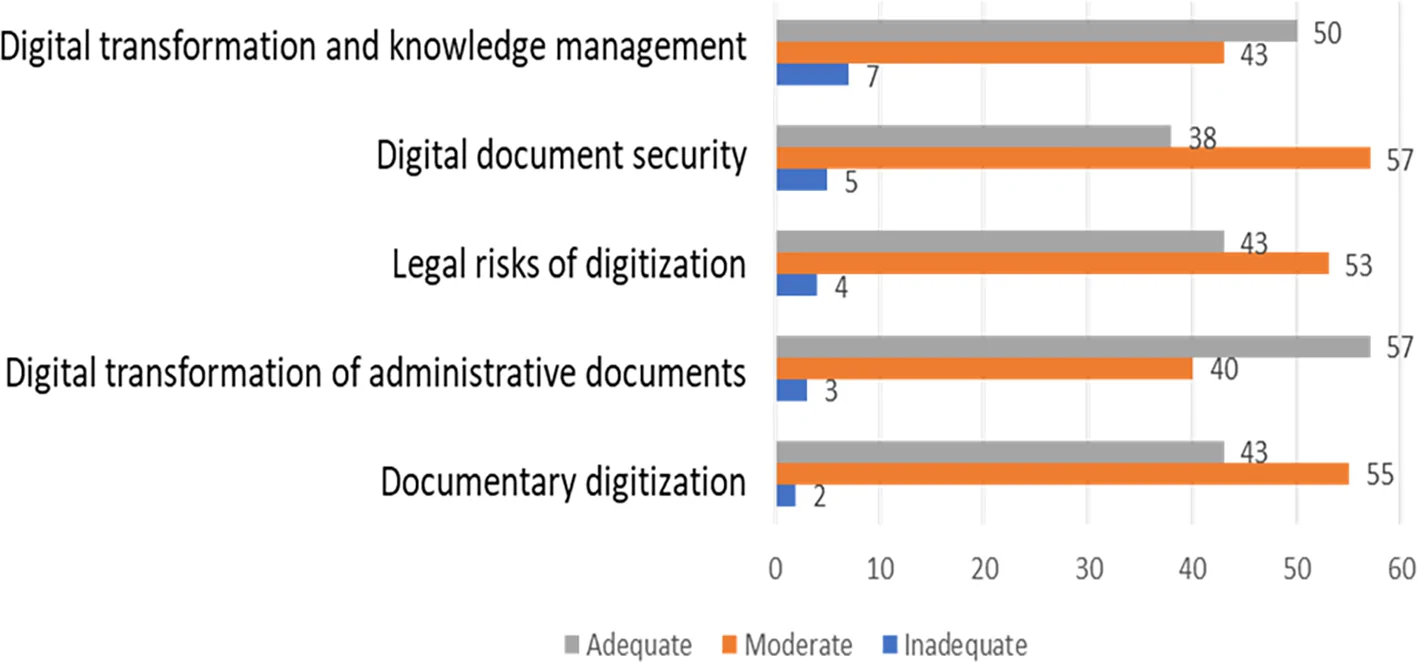
Level of use of document digitization as reported by administrative staff at a municipality in the province of Utcubamba, Amazonas Department, Peru.

**Figure 2.**
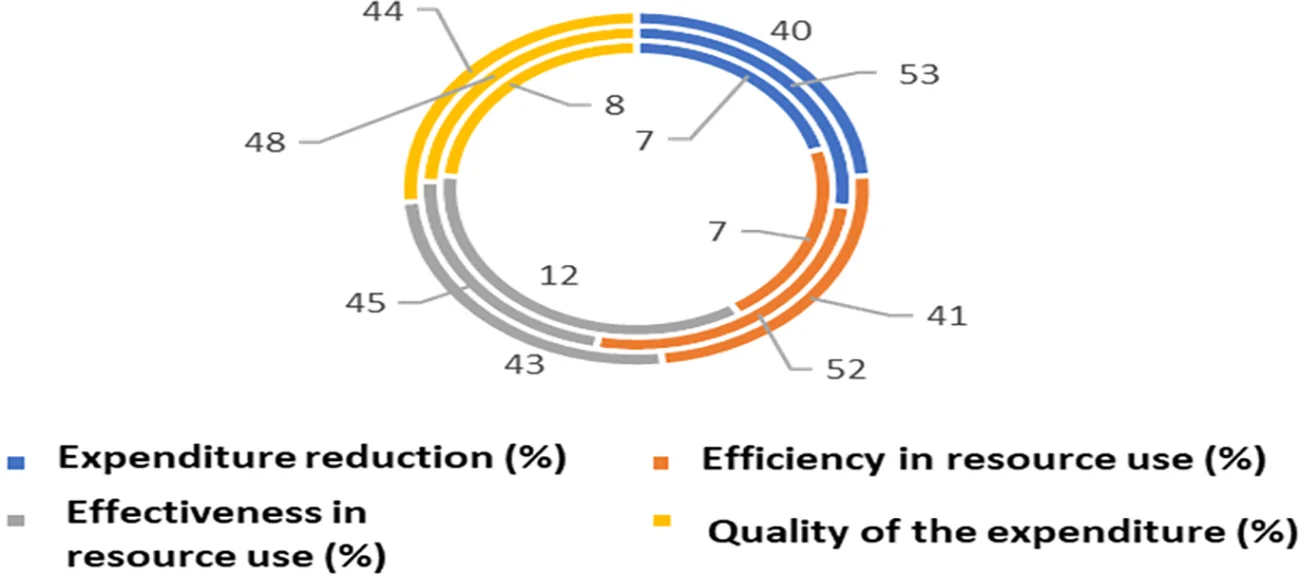
Workers’ Perceptions of Resource Management and the Quality of Municipal Spending.

### Statistical analysis

#### Multiple linear regression: perceptions of resource management or quality of spending as an outcome

One a priori model containing four predictors—digital transformation, legal risks, security, and knowledge management—was tested (N = 95). Model fit and inference are reported once and are used consistently throughout (prior exploratory summaries producing different R/R ^2^ values were deprecated and are not reported here to avoid inconsistency).

### Model summary

Table 1 shows that, for the 95 employees who completed the survey, the four dimensions of document digitization were significantly related to perceptions regarding the use of resources, the achievement of objectives, and the quality of municipal spending (R ^2^ = 0.763). This value indicates the proportion of variance in the observed scores in the sample, not the percentage of savings in expenditures. When the four dimensions were introduced simultaneously, and using HC3 standard errors, the scores corresponding to the legal aspects of digitization (B = 0.374; p = 0.010) and to digital transformation and knowledge management (B = 0.356; p = 0.015) continued to show a positive association. The digital transformation of administrative documents was not significantly associated (p = 0.920). Document security showed a positive coefficient, but the HC3 interval included zero (p = 0.059), so there is no conclusive evidence in this regard using our primary method of inference. These associations should be interpreted with caution given the nature of the instrument. The “legal aspects” dimension also includes questions on planning, processes, and training. Similarly, the dimension on digital transformation and knowledge management combines statements on citizen participation, digital tools, and administrative performance. Therefore, their coefficients do not allow us to identify the contribution of a specific legal or knowledge management mechanism.

**Table 1.** Multiple linear regression of the dimensions of document digitization and their association with perceptions of efficiency, effectiveness, and quality in the use of municipal resources.

Predictor	B	EE HC3	β	t HC3	p HC3	IC 95% HC3 for B
Constant	−0.110	0.280	-	−0.395	0.694	[−0.666;0.446]
Digital transformation of administrative documents	0.011	0.113	0.009	0.100	0.920	[−0.213;0.236]
Legal risks of digitization	0.374	0.143	0.331	2.620	0.010	[0.090;0.658]
Document security	0.277	0.145	0.271	1.915	0.059	[−0.010;0.565]
Digital transformation and knowledge management	0.356	0.143	0.328	2.480	0.015	[0.071;0.640]

*Note:* N = 95. The dependent variable is the average of the 42 items regarding perceptions of resource use, goal achievement, and quality of spending. B = unstandardized coefficient; EE HC3 = HC3 robust standard error; β = standardized coefficient; CI = confidence interval. Model fit: R = 0.873; R ^2^ = 0.763; adjusted R ^2^ = 0.752; F(4, 90) = 72.325; p < 0.001. The individual tests and intervals in the table use HC3 standard errors.

Supplementary Figure S1 points out that we analyzed the plots of residuals versus fitted values and the Q–Q plots to assess the model’s shape and the dispersion of the residuals. The RESET test for the null hypothesis that the specified functional form is correct was not rejected: F(2, 88) = 0.160, p = 0.853. Both the Breusch-Pagan test, χ ^2^(4) = 3.092, p = 0.543, and the White test, χ ^2^(14) = 22.564, p = 0.068, suggest that there is no statistically significant evidence, at the 0.05 level, of heteroscedasticity. The Shapiro-Wilk tests indicated that the residuals did not follow a normal distribution (W = 0.955, p = 0.003). The variance inflation factors were 2.10 for the digital transformation of administrative documents, 3.51 for the legal risks of digitization, 4.16 for document security, and 4.49 for digital transformation and knowledge management. Nine observations fell outside the exploratory threshold of Cook’s distance of 4/95; the maximum value was 0.409. These observations were retained in the main analysis. HC3 standard errors were used for coefficient estimation, taking into account the residual diagnosis and the potential impact of individual observations.

Supplementary Figure S2 points out that in a sensitivity analysis using 5,000 nonparametric samples obtained via the bootstrap method, the 95th percentile confidence intervals for the risks associated with digitization [0.107; 0.620] and with digitization and knowledge management [0.069, 0.624] did not include zero. Regarding document security, the bootstrap confidence interval [0.011, 0.548] excluded zero, but its HC3 confidence interval from Table 1 [−0.010; 0.565] did include it. Therefore, the results regarding security depend on the test used, and the main conclusions are based on the pre-specified HC3 results. The Durbin-Watson test was not used for these cross-sectional responses, as the sequence of questionnaire records has no temporal or spatial relevance.

## Discussion and conclusion

The study analysed the correlation between (i) four dimensions related to document digitization and the perceptions of administrative office workers regarding resource consumption, goal achievement, and the quality of municipal spending. Among the 95 questionnaires analysed, the combined model had an R ^2^ of 0.763. This value represents the amount of variance in the scores reported by participants that is explained by the four predictors in this sample; it was not equivalent to budget savings nor did it indicate that digitization would have led to a reduction in spending. When controlling for all four dimensions simultaneously and using HC3 standard errors, the variables “legal risks of digitization” and “digital transformation and knowledge management” were statistically significantly and positively associated with the outcome. In contrast, the digital conversion of administrative documentation was not statistically significantly associated with any other factor. The security of digital documents yielded a positive coefficient, but its confidence interval included zero, and its p-value was 0.059. Consequently, under the adopted inference procedure, the results do not allow us to conclude that there is a conclusive statistical association for this last dimension.

These results help refine the theoretical framework. Androniceanu y Georgescu (2023) analysed the digitization of public administration in relation to government performance in European Union member states. Their research helps situate digitization within a broader debate on institutional performance. In this study, the findings are significant for the dimensions that include procedures and knowledge management, while the fourth dimension—document digitization—when considered in conjunction with the others, does not yield any statistically identifiable association. The studies vary in context, unit of analysis, and measurements; therefore, this comparison is conceptual and does not constitute a direct transfer of their results to Peruvian municipalities.

The conceptual framework argues that the availability of various organized and accessible documents can facilitate administrative processes and resource management. Hagen (2023) indicates, through a qualitative study, that digitization presents both challenges and opportunities for service delivery and user support in institutions of varying sizes, and that these institutions have different capabilities. This finding clarifies that the mere presence of digital tools in an institution may result in a partial representation of how the institution operates. However, our survey did not ask directly about the time spent searching for materials, productivity levels, document preservation, or archiving costs; these factors cannot be presented as findings of this study.

The positive association between the transformational digital dimension and knowledge management is consistent with the idea that the organization and exchange of information could be accompanied by a more positive assessment of administrative performance. However, it should be noted that this dimension combines a variety of elements, including digital tools, training, and citizen participation. Androniceanu y Georgescu (2022) focus on e-participation in Europe; this article provides background information for understanding this aspect of the dimension but does not demonstrate that e-participation reduces costs for the municipality under study. The variety of content also suggests the need to explore the scale’s factor structure more thoroughly in future studies.

With regard to the dimension of the so-called legal risks of digitization, its positive coefficient should be interpreted by taking into account the items that comprise it. This dimension consists of a set of opinions on regulatory and organizational aspects; the observed association does not imply that litigation, penalties, or legal expenses have decreased. One possible explanation is that employees who perceive procedures as being clearer are also those who hold a more favourable opinion of resource management. This line of reasoning would need to be verified using the organization’s own administrative indicators.

### Scope of the findings and implications

The results are based on 95 workers who responded out of 125 invited participants in a municipality. No stratified random sampling was conducted, and with the available information, it has not been possible to determine to what extent the 30 non-responding workers differ from the participants. Job duties, training, the availability of systems, and conditions in each area could be associated with both experiences of digitization and performance evaluations. The cross-sectional study design cannot determine the temporal order of these relationships or causality. The study’s contribution is to identify, within this institutional framework, which dimensions remain related to perceptions of resource management when evaluated together. The results provide grounds for investigating the clarity of knowledge management procedures and practices. They are not evaluations of the financial performance of digitization projects, nor do they constitute a sufficiently solid basis for recommending investments in infrastructure or specific technologies.

### Limitations (method-aware)

This study has limitations in terms of design, measurement, and analysis. Because this is a cross-sectional survey, the observed associations do not allow us to draw conclusions about whether digitization practices preceded perceptions of resource management or whether there is a causal relationship between the two. All variables were derived from responses provided by the same employees at a single point in time; therefore, common-method bias may also have exaggerated the associations. No statistical test allows us to definitively rule out that possibility. The dependent variable was constructed based on questions regarding resource use, the achievement of objectives, and the quality of spending. We did not analysed accounting records, budgeted amounts, or the processing times for sales opportunities. Therefore, the study does not quantify actual savings in municipal expenditures. Furthermore, if some items in the predictor dimensions refer to resources or paper, there may be a conceptual proximity to the outcome. The high internal consistency of the scales does not in itself indicate that their dimensions are distinct constructs. The interpretation of document security should be approached with particular caution. Although its coefficient was positive, the confidence interval (based on HC3 standard errors) included zero (p = 0.059). The conclusion regarding its significance depends on the choice of method for estimating the standard error; therefore, this dimension cannot be considered an unambiguously significant predictor. It is possible that other uncontrolled characteristics in the model—such as the worker’s job duties, training, or the technological conditions of their unit—may also have had an influence. These possibilities limit the interpretation of the estimated coefficients.

***Scope***

A total of 125 administrative employees of the city government were invited to participate, and 95 of them (76.0% of those invited) responded. There is no documented comparison between participants and non-participants to assess nonresponse bias. The results refer to responses from a single institution and are not necessarily representative of all Peruvian municipalities. Differences among local governments in terms of budget, connectivity, volume of documents, staff, and administrative structure can affect both the implementation of digitization and its relationship to performance. Future studies should analyze whether similar associations exist in other municipalities. Similarly, the theoretical framework provided by the work of Androniceanu and Georgescu (2023) on government effectiveness in European countries and by Hagen (2023) on records management in Norwegian institutions is relevant, but the results cannot be directly generalized to this municipality.

***Future research***

Future studies could cover several municipalities and utilize both questionnaires and actual records of budget execution, processing times, paper consumption, and document preservation costs. A “before-and-after” intervention, with appropriate institutional oversight, would allow for a more direct verification of the causal chain proposed in the theoretical framework between digitization, changes in administrative processes, and potential changes in spending.

***Practical implications (non-causal, decision-useful)***

The fact that the dimensions of legal risks, digital transformation, and knowledge management showed positive associations suggests that it is worthwhile to analyze how procedures are documented, how responsibilities are communicated, and how access to information is structured within municipal institutions. An assessment of the institution may reveal specific obstacles before deciding to make a change in document management, training, or coordination among units. The survey does not imply that such actions will generate savings, as their usefulness and cost must be evaluated separately.

The fact that the document did not reveal a statistically robust association with security does not serve as an excuse for failing to protect information. Policies regarding access control, backup, and retention must take into account the institution’s document management needs and risks. Similarly, new systems or specific technologies should only be considered in light of their suitability for current processes, their implementation and maintenance costs, and the municipality’s capacity to manage them. To determine whether a measure truly reduces expenses, the municipality should establish performance indicators for the measure and then compare them with verifiable data, such as printing and storage costs, processing times, and associated budget line items. Such an evaluation would make it possible to distinguish between improvements “felt” by staff and observed administrative and financial changes.

## Conclusion

In the 95 questionnaires, the dimensions of legal risks in digitization and digital transformation and knowledge management were positively associated with employees’ perceptions regarding the use of resources, the achievement of goals, and the quality of municipal spending, after controlling for the other dimensions of the model. No additional statistically significant association was detected for the digital transformation of administrative documents. Nor was the estimated positive effect for document security conclusive when HC3 standard errors and a significance level of 0.05 were used. The findings suggest that the organizational and regulatory aspects of digitization could also warrant attention in the municipality under study. However, the cross-sectional study does not allow for the establishment of causal relationships, and the dependent variable does not measure expenditure amounts. Consequently, the research does not prove actual reductions in paper, storage, or labor costs, nor does it allow for the measurement of municipal savings. Its results refer to the responses of a single institution and need to be verified before being applied to other local governments.

As a next step, the municipality could analyze its document management processes, training needs, and knowledge organization, taking into account the costs and feasibility of implementing the suggested changes. To investigate whether digitization helps reduce expenses, future studies should integrate staff perceptions with accounting data and process indicators measured over time and, if possible, include other municipalities in the analysis.

## Ethics and consent

Ethical approval for the project “Public Documents Digitization: Towards Efficiency and Reduction of Municipal Expenses” was granted by the Comité de Ética en Investigación de Contabilidad – Pregrado, Universidad César Vallejo (UCV), Chiclayo, Perú. The committee verified the protocol, instruments, informed-consent procedures, and authorization to conduct the study in its Reporte de Verificación de Requisitos dated 19 July 2024.

Institutional approval of the research project was subsequently issued by UCV through Resolution No. 051–2024-UCV-VA-CON-F02/CEP (Chiclayo, 26 July 2024), which serves as the formal ethics approval/reference number for this study.

UCV does not assign a separate standalone IRB/ethics code beyond the committee’s verification and the university resolution; therefore, no additional approval ID is available. Written informed consent was obtained from all participants prior to data collection; all respondents were ≥ 18 years old. The study adhered to the Declaration of Helsinki and to applicable national and institutional research ethics standards. No independent external review board participated; ethics oversight was provided internally by UCV’s committee.

## Additional materials and data

The database, the complete questionnaire, the adjusted regression tables, the residuals-versus-adjusted-values and Q–Q plots, and the Python script used for statistical diagnostics are available in the same B2SHARE repository: https://doi.org/10.23728/b2share.w5xx7-krz29 ( Condor, M. M. C. 2025).

## Process identifier

These materials allow for open replication and review of the study. The complete dataset is accessible without restrictions or embargoes under a CC-BY 4.0 licence.

## Data availability

All data supporting the results of this study, including the values underlying the means, standard deviations, and other reported measures; the values used to generate the figures; and the data points extracted from the images for analysis, are available in Segundo Cajusol’s data availability repository.

### Excel database

**B2SHARE EUDAT: DOI**: https://doi.org/10.23728/b2share.w5xx7-krz29 ( Condor, M. M. C. 2025).
