# Supplementary material for: Public Documents Digitization: Towards Efficiency and Reduction of Municipal Expenses
Source: F1000Res. 2026 Nov 4;14:918. Originally published 2025 Sep 12. [Version 4] doi: 10.12688/f1000research.169678.4 (PMC13740457; doi:10.12688/f1000research.169678.4)
Supplement: Peer review history

# Reviewer report: Reviewer response for version 4

**DOI:** 10.5256/f1000research.211363.r538036

**Published:** 2026-11-05

**Authors:** Irina Georgescu

**Affiliations:** 1 Bucharest University of Economics, Bucharest, Romania

The authors have substantially improved the manuscript  I consider the revised article broadly satisfactory.

Is the work clearly and accurately presented and does it cite the current literature?

Yes

If applicable, is the statistical analysis and its interpretation appropriate?

Partly

Are all the source data underlying the results available to ensure full reproducibility?

Yes

Is the study design appropriate and is the work technically sound?

Partly

Are the conclusions drawn adequately supported by the results?

Partly

Are sufficient details of methods and analysis provided to allow replication by others?

Yes

Reviewer Expertise:

digital transformation; machine learning applications in economics; public policy evaluation.

I confirm that I have read this submission and believe that I have an appropriate level of expertise to confirm that it is of an acceptable scientific standard.

# Reviewer report: Reviewer response for version 3

**DOI:** 10.5256/f1000research.208039.r516555

**Published:** 2026-09-25

**Authors:** Andi Cudai Nur

**Affiliations:** 1 Makassar State University, Makassar, Indonesia

The background section requires revision to emphasize that the analysis demonstrates a significant positive impact of document digitization on the operational costs of the municipal government offices studied—driven primarily by reduced paper usage, lower storage costs, and faster administrative processes. The methodology section needs to further elaborate on the regression and variance analysis results, which indicate that this impact is uneven across various elements of digital administration; specifically, elements related to knowledge of legal risk management and knowledge management possess greater explanatory power than the mere implementation of the technology itself. This suggests that cost reductions are driven more by how digitization is managed than by the technology implementation itself.

 The discussion section—which links the problem statement, data analysis results, and research findings—requires a more structured and operationally rational approach; the results must reinforce evidence of persistent operational shortcomings. Although overall effectiveness indicators show positive results, the findings reveal a neutral impact from two crucial processes—information security and legal compliance—which limit the realization of maximum financial benefits from digitization. By highlighting empirical patterns consistent with prior findings in similar public sector contexts, this study offers valuable insights that can strengthen the conclusion that partial or fragmented digitization approaches yield only limited efficiency gains rather than comprehensive cost savings.

 We, as reviewers, have no conflicts of interest regarding the review of this manuscript.

Is the work clearly and accurately presented and does it cite the current literature?

Yes

If applicable, is the statistical analysis and its interpretation appropriate?

Yes

Are all the source data underlying the results available to ensure full reproducibility?

Yes

Is the study design appropriate and is the work technically sound?

Yes

Are the conclusions drawn adequately supported by the results?

Yes

Are sufficient details of methods and analysis provided to allow replication by others?

Yes

Reviewer Expertise:

Public Administration

I confirm that I have read this submission and believe that I have an appropriate level of expertise to confirm that it is of an acceptable scientific standard.

# Reviewer report: Reviewer response for version 3

**DOI:** 10.5256/f1000research.208039.r516556

**Published:** 2026-09-03

**Authors:** Irina Georgescu

**Affiliations:** 1 Bucharest University of Economics, Bucharest, Romania

The manuscript contains serious methodological and reporting problems:

 1. The Methods report 92 completed questionnaires, whereas the regression uses N = 95. The degrees of freedom also correspond to 95 observations. 

 2. The requested analyses remain incomplete. There is no common-method-bias test, factor analysis, or properly reported robustness analysis.

 3. The manuscript contains unfinished placeholders and editing errors, such as “Table X,” “repository name blinded,” “applied to the patients,” “BBB,” and “2.5 [p9].”

 4. The manuscript incorrectly places Iquitos in the Amazonas region, although Iquitos is in Loreto.

 5. The conclusion contradicts the regression results. It claims an overall significant effect of digitization and actual expenditure reductions, although overall digital transformation is nonsignificant and expenditure reduction is measured through employee perceptions.

Is the work clearly and accurately presented and does it cite the current literature?

Yes

If applicable, is the statistical analysis and its interpretation appropriate?

Partly

Are all the source data underlying the results available to ensure full reproducibility?

Yes

Is the study design appropriate and is the work technically sound?

Partly

Are the conclusions drawn adequately supported by the results?

Partly

Are sufficient details of methods and analysis provided to allow replication by others?

Yes

Reviewer Expertise:

digital transformation; machine learning applications in economics; public policy evaluation.

I confirm that I have read this submission and believe that I have an appropriate level of expertise to state that I do not consider it to be of an acceptable scientific standard, for reasons outlined above.

# Response

**Published:** 2026-09-24

**Authors:** María Maricely Chuquilin Condor

**Affiliations:** LA LIBERTAD, Universidad Cesar Vallejo, Trujillo, La Libertad, Peru

Good evening, dear reviewer.

 Thank you very much for taking the time to review my work. Please be advised that we have addressed the comments.

 We look forward to hearing from you.

 With gratitude

# Reviewer report: Reviewer response for version 2

**DOI:** 10.5256/f1000research.195979.r502475

**Published:** 2026-07-21

**Authors:** Irina Georgescu

**Affiliations:** 1 Bucharest University of Economics, Bucharest, Romania

This study examines the relationship between public document digitization and municipal expenditure reduction using survey data collected from administrative staff in a municipality in the Amazonas region of Peru. The results indicate that legal risk management and knowledge management are the strongest dimensions associated with perceived cost reduction. Overall digital transformation and document security are not statistically significant after controlling for the other dimensions. The findings suggest that organizational and governance practices contribute more to administrative efficiency than the mere adoption of digital technologies.

 The main novelty lies in providing empirical evidence from a relatively understudied Peruvian municipal context while demonstrating that legal compliance and knowledge management are more strongly associated with municipal cost reduction than digital technology adoption alone.

**Weaknesses:**

 1. Expand the discussion of external validity and acknowledge that findings from a single municipality cannot be readily generalized to other municipalities or countries.

 2. Address the potential for common method bias by reporting an appropriate diagnostic test (e.g., Harman's single-factor test) or discussing this limitation.

 3. Strengthen the statistical analysis by including additional robustness or sensitivity analyses (e.g., bootstrapping, alternative model specifications, or validation procedures).

 4. Provide stronger evidence for construct validity by conducting and reporting exploratory or confirmatory factor analysis in addition to internal consistency measures.

 5. Discuss the limitations of using perceived expenditure reductions.

 6. Moderate the policy recommendations so that they remain aligned with the empirical evidence. Distinguish evidence-based findings from broader practical implications.

 7 Refer to papers: [REF1 & 2]

Is the work clearly and accurately presented and does it cite the current literature?

Yes

If applicable, is the statistical analysis and its interpretation appropriate?

Partly

Are all the source data underlying the results available to ensure full reproducibility?

Yes

Is the study design appropriate and is the work technically sound?

Partly

Are the conclusions drawn adequately supported by the results?

Partly

Are sufficient details of methods and analysis provided to allow replication by others?

Yes

Reviewer Expertise:

digital transformation; machine learning applications in economics; public policy evaluation.

I confirm that I have read this submission and believe that I have an appropriate level of expertise to state that I do not consider it to be of an acceptable scientific standard, for reasons outlined above.

## References

[1] : Public Administration Digitalization and Government Effectiveness in EU Countries. *Central European Public Administration Review* .2023;21(1) : 10.17573/cepar.2023.1.017-30 10.17573/cepar.2023.1.01

[2] : E-PARTICIPATION IN EUROPE: A COMPARATIVE PERSPECTIVE. *Public Administration Issues* .2022; 10.17323/1999-5431-2022-0-5-7-297-29 10.17323/1999-5431-2022-0-5-7-29

# Response

**Published:** 2026-08-04

**Authors:** María Maricely Chuquilin Condor

**Affiliations:** LA LIBERTAD, Universidad Cesar Vallejo, Trujillo, La Libertad, Peru

We appreciate this important observation. We agree that conducting the study in a single municipality limits the direct generalizability of the results. In response, we have expanded the contextual scope of the study and the discussion of its external validity. We now clarify that, although the probabilistic sampling allowed us to represent the administrative areas of the studied entity, the sample does not represent all Peruvian municipalities or local governments in other countries.

 We have also included an explanation of the factors that could influence the transferability of the findings, including fiscal capacity, technological infrastructure, level of urbanization, connectivity, digital maturity, organizational complexity, and the regulatory framework. We have also qualified our conclusions and proposed comparative and multicenter studies to assess the stability of these associations in other contexts.

 Revisions Made

 The discussion on external validity was expanded, the limitations section was reworded, the conclusions were tempered, and recommendations for future multicenter research were incorporated.

# Reviewer report: Reviewer response for version 1

**DOI:** 10.5256/f1000research.187036.r420696

**Published:** 2025-12-04

**Authors:** Andi Cudai Nur

**Affiliations:** 1 Makassar State University, Makassar, Indonesia

While this work is presented clearly and accurately, explanations are still needed for all variables and indicators studied in the tables and figures. This work has cited the latest literature, and the research design is sound, but it needs to be more technically structured for ease of understanding. The details of the methods and analysis provided are adequate, but still require clarity and supporting arguments, preferably in line with the data analysis for "digitizing public documents, efficiency, and reducing local government spending" to allow for replication by others.

 The statistical analysis and its interpretation in the tables and figures require more precise explanations according to the variables and indicators studied, and the results of each data analysis for: digitizing public documents, efficiency, and reducing local government spending, must be written in a structured manner.

 The conclusions drawn must be adequately supported by the results of the data analysis for each variable and indicator studied and must be written in a complete and more structured manner.

Is the work clearly and accurately presented and does it cite the current literature?

Yes

If applicable, is the statistical analysis and its interpretation appropriate?

Yes

Are all the source data underlying the results available to ensure full reproducibility?

Yes

Is the study design appropriate and is the work technically sound?

Yes

Are the conclusions drawn adequately supported by the results?

Yes

Are sufficient details of methods and analysis provided to allow replication by others?

Yes

Reviewer Expertise:

Public Administration

I confirm that I have read this submission and believe that I have an appropriate level of expertise to confirm that it is of an acceptable scientific standard, however I have significant reservations, as outlined above.

# Reviewer report: Reviewer response for version 1

**DOI:** 10.5256/f1000research.187036.r420693

**Published:** 2025-10-15

**Authors:** Isagani M. Tano

**Affiliations:** 1 Quezon City University, Quezon City, Philippines

The authors investigate the relationship between document digitization and cost reduction in a public municipal organization in Amazonas, Peru, using a quantitative, cross-sectional survey of administrative staff. They sampled 95 respondents out of a population of 125 administrative workers, administered a 90-item questionnaire, and analyzed data with descriptive and inferential statistics (regression and ANOVA) using SPSS v27. The paper reports high instrument reliability (Cronbach’s α ≈ 0.91) and finds that components of digitization — particularly legal risk management and knowledge management — are positively associated with cost reduction; reported model fit statistics indicate that digitization variables explain a substantial portion of variance in cost outcomes. The authors present policy recommendations on security, infrastructure, training, and knowledge systems, and publish the instrument and dataset in a public repository.

 The topical and policy-relevant question of the digitization of municipal documents is a timely topic with clear practical implications for cost savings and governance.  In good data-sharing practice, the authors made the dataset and instruments publicly available (B2SHARE DOI), which supports reproducibility.  Adequate sample proportion sampling of 95/125 administrative staff gives reasonable statistical power for many analyses if sampling and response processes are sound.  High reported internal consistency (Cronbach’s α reported (~0.912, 0.916) indicates reliable scales.

 A. Inconsistent and unclear statistical reporting (must be corrected).

 Problem: The manuscript reports conflicting model statistics in different parts of the text/tables: e.g., Table 1 lists R = 0.816 and R² = 0.666, Table 4 lists R = 0.758 and R² = 0.575, and elsewhere the discussion mentions F = 125.987 and r = 11.224 (which do not match table values). P-values are sometimes formatted as “,000”. These inconsistencies undermine confidence in the results.

 Fix (required): Recompute and carefully verify all model outputs. Ensure one consistent set of values is presented across text, tables, and figures. Use standard formatting (e.g., p < 0.001). Provide complete regression tables including: unstandardized B, SE(B), standardized β, t, p, 95% confidence intervals for coefficients, model F, df, R, R², and adjusted R². Correct any typographical or numeric errors.

 B. Missing model diagnostics and robustness checks (must be added).

 Problem: No diagnostic tests are reported (residual plots, tests for linearity, heteroscedasticity, normality, leverage/influential points), and no checks for multicollinearity (VIF/Tolerance) are shown. Without these, regression assumptions are unverified.

 Fix (required): Add diagnostics: residual vs fitted plot, Q–Q plot, Breusch–Pagan test (or White test) for heteroscedasticity, Durbin–Watson for autocorrelation (if applicable), VIF for each predictor, and influence statistics (Cook’s D). If assumptions fail, state remediation (e.g., robust SE, transformation, or alternative models).

 C. Insufficient detail on sampling and response (must be clarified).

 Problem: The paper states stratified probability sampling and inclusion criteria but lacks details about strata definitions, how respondents were selected within strata, response rates, and whether any nonresponse bias analysis was performed.

 Fix (required): Provide: (1) the exact stratification variables and counts in population and sample per stratum, (2) sampling procedure (random draw, systematic, etc.), (3) response rate and how many refused or were excluded, and (4) any weighting applied. If possible, include a CONSORT-like flow diagram of participant selection.

 D. Replication details partially missing (must be completed).

 Problem: The text mentions a 90-item questionnaire validated by experts and high Cronbach’s alpha, and points to the data repository — but the manuscript should explicitly state the mapping of questionnaire items to constructs, scoring rules, scale ranges, and any reverse-coding performed.

 Fix (required): In the Methods or Supplementary Material: include the instrument as Appendix (if not already present in the repository), the codebook with variable names, response options, item-to-scale mappings, scoring algorithm, and SPSS syntax or R script used for analyses. Indicate where in the repository these files are located.

 E. Interpretation and causal language need to be tempered (should be revised).

 Problem: The cross-sectional design supports associations, but parts of the discussion imply causality (“exerts a significant influence”).

 Fix (required): Reword conclusions to reflect associative findings and explicitly state limitations regarding causal inference. Discuss plausible confounders and alternative explanations. Recommend longitudinal or quasi-experimental designs for causal claims.

 F. Presentation and typographical issues (should be fixed).

 Problem: Several formatting and language issues (comma vs decimal formatting, stray sentence fragments, figure/table numbering mismatches) distract from readability. Examples include inconsistent decimal separators and mixed English/Spanish punctuation and occasional fragmented sentences.

 Fix (requested): Thorough copyedit for language, consistent numeric formatting (use period as decimal separator for English manuscripts), accurate figure/table references, and consistent statistical notation.

 Confidence intervals and effect sizes: Add 95% CIs for key parameters and consider reporting standardized effect sizes to help assess practical significance.  Model selection rationale: Explain why predictors were chosen and whether any variable selection method was used.  Control variables: If available, include potential confounders (age, tenure, department, prior digitization exposure) in adjusted models; if not measured, state that as a limitation.  The internal ethics committee and university resolution are reported; consider clarifying whether local municipal permissions were required/obtained beyond institutional approval. Ensure all figures are high resolution and that axes and legends are self-contained. Consider adding a schematic of the hypothesized causal pathway. Tighten the policy recommendations by linking each suggested action (e.g., PKI, blockchain) to feasibility and cost relative to municipal budgets or frame them as options to be evaluated further rather than firm prescriptions.

 Resolve inconsistencies in reported statistics across text/tables (this is essential).  Provide full regression output with CIs and SEs and uniformly formatted p-values.  Add model diagnostics and address assumption violations (or report robust alternatives).  Clarify sampling and response procedures with stratum counts and response rates. Include analytic scripts or exact commands used (SPSS syntax or R scripts) in the repository and reference them in the manuscript for true reproducibility.

Is the work clearly and accurately presented and does it cite the current literature?

Partly

If applicable, is the statistical analysis and its interpretation appropriate?

Partly

Are all the source data underlying the results available to ensure full reproducibility?

Yes

Is the study design appropriate and is the work technically sound?

Partly

Are the conclusions drawn adequately supported by the results?

Partly

Are sufficient details of methods and analysis provided to allow replication by others?

Partly

Reviewer Expertise:

Public administration and digital governance; quantitative evaluation methods in social sciences (survey design, regression analysis, reproducible data practices).

I confirm that I have read this submission and believe that I have an appropriate level of expertise to confirm that it is of an acceptable scientific standard, however I have significant reservations, as outlined above.
